# The effect of COVID-19 on cancer incidences in the U.S

**DOI:** 10.1016/j.heliyon.2024.e28804

**Published:** 2024-03-30

**Authors:** Ramalingam Shanmugam, Larry Fulton, C. Scott Kruse, Brad Beauvais, Jose Betancourt, Gerardo Pacheco, Rohit Pradhan, Keya Sen, Zo Ramamonjiarivelo, Arvind Sharma

**Affiliations:** a Texas State University, School of Health Administration, Encino Hall, Room 250A, 601 University Drive, San Marcos, TX, 78666, USA; b Boston College, Woods College of Advancing Studies, St. Mary's Hall South, Chestnut Hill, MA, 02467, USA

**Keywords:** Principal components analysis, Pre, During, And post COVID-19 indices, Most likely cancer incidence, Radar plot, Angles, Indices

## Abstract

Fundamental data analysis assists in the evaluation of critical questions to discern essential facts and elicit formerly invisible evidence. In this article, we provide clarity into a subtle phenomenon observed in cancer incidences throughout the time of the COVID-19 pandemic. We analyzed the cancer incidence data from the American Cancer Society [1]. We partitioned the data into three groups: the pre-COVID-19 years (2017, 2018), during the COVID-19 years (2019, 2020, 2021), and the post-COVID-19 years (2022, 2023). In a novel manner, we applied principal components analysis (PCA), computed the angles between the cancer incidence vectors, and then added lognormal probability concepts in our analysis. Our analytic results revealed that the cancer incidences shifted within each era (pre, during, and post), with a meaningful change in the cancer incidences occurring in 2020, the peak of the COVID-19 era. We defined, computed, and interpreted the exceedance probability for a cancer type to have 1000 incidences in a future year among the breast, cervical, colorectal, uterine corpus, leukemia, lung & bronchus, melanoma, Hodgkin's lymphoma, prostate, and urinary cancers. We also defined, estimated, and illustrated indices for other cancer diagnoses from the vantage point of breast cancer in pre, during, and post-COVID-19 eras. The angle vectors post the COVID-19 were 72% less than pre-pandemic and 28% less than during the pandemic. The movement of cancer vectors was dynamic between these eras, and movement greatly differed by type of cancer. A trend chart of cervical cancer showed statistical anomalies in the years 2019 and 2021. Based on our findings, a few future research directions are pointed out.

## 1 Introduction

The COVID-19 pandemic may have had secondary and tertiary effects on cancer screening and the incidence of cancer itself [1]. Public health professionals ponder whether these incidence rates are an exception attributable to the impact of COVID-19, or rather because of other exogenous factors. With this as our defined research aim, we intend to answer the following research question: Are the patterns of major cancer incidences same in the United States (U.S.) in pre- (in years 2017, 2018, and 2019), during- (in years 2020, 2021), and post- (in years 2022, 2023) eras of the COVID-19? For this purpose, using national cancer data from the American Cancer Society [2], we pursue an analytic approach in this article to trace whether any shift in cancer incidence has occurred due to COVID-19. An innovative angular index is introduced to rank the cancers in the epochs of above defined time.

The National Cancer Institute defines cancer as “a disease in which some of the body's cells grow uncontrollably and spread to other parts of the body” [3]. The treatment of skin cancer was delayed in the United Kingdom due to the pandemic. Also, the treatment rates were calculated for the years 2017 through 2023 [4]. The cancer treatment in Australia were postponed as well in pre-, during-, and post- COVID-19 era [5]. The COVID-19 pandemic disrupted the cancer detection and treatment in Ontario, Canada [6]. Tobacco is known to be a cancer-causing substance. Other proven causes of cancer are genetic mutations or environmental factors [7]. The American Cancer Society reported that the number of new cancer cases in the United States declined during 2019 and 2020 (by 1.5%), during the pandemic era [7]. This delay could cause an upward shift in the prevalence data because incidence was not immediately treated.

In a recent article, Shanmugam et al. [8] developed a stochastic model for the number of COVID-19 fluctuating fatalities across United States (U.S.) counties. The COVID-19 was the third leading cause of death in the U.S. behind heart disease and cancer during 2019 and 2020. Some health professionals noticed a tendency of over counting the COVID-19 deaths while others, on the contrary, indicated delayed reporting of cancer cases during the COVID-19. The de-confounding of the treatment delay or the cancer as cause of death was really the issue [[9], [10], [11], [12]].

**Fig. 1:**
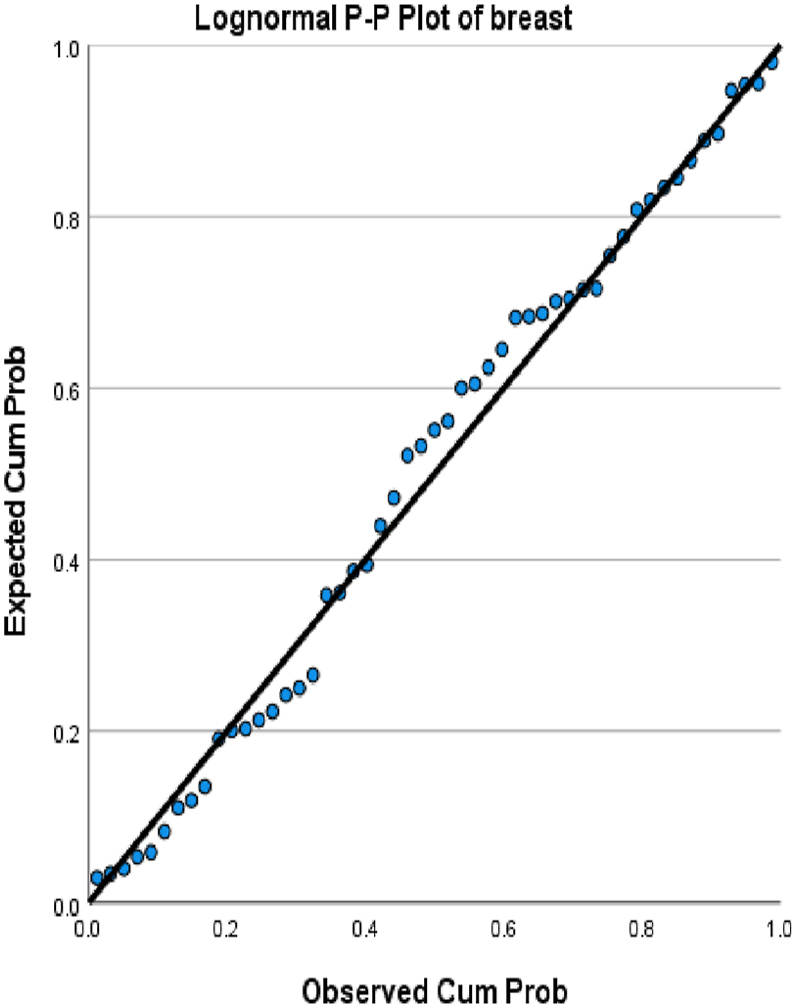
Breast cancer.

**Fig. 2:**
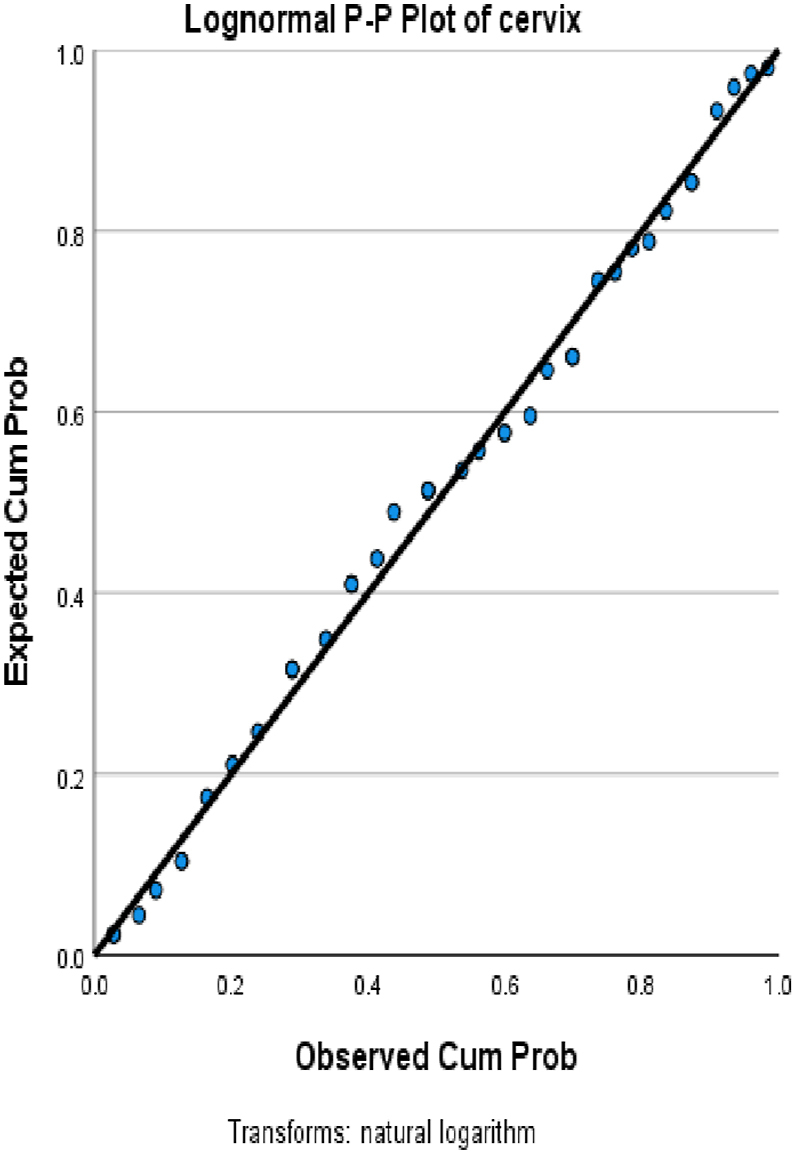
Cervical cancer.

**Fig. 3:**
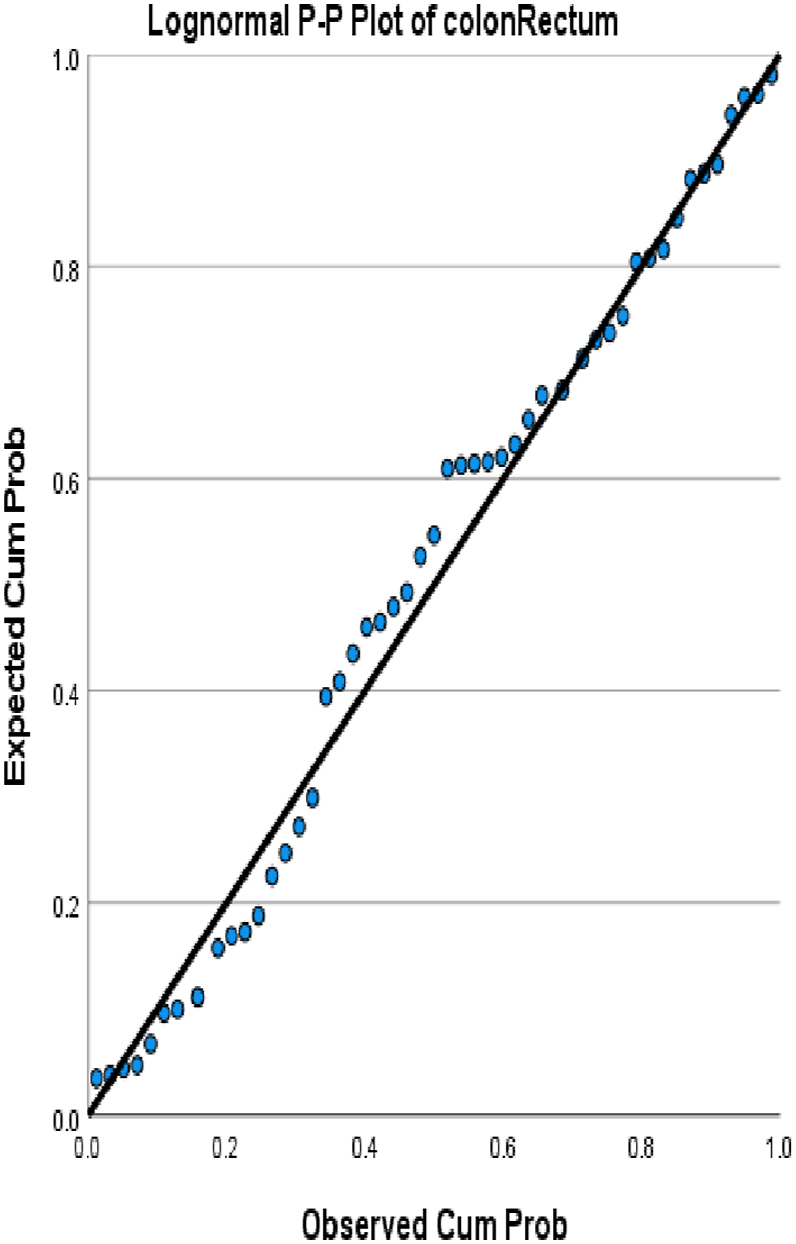
Colorectal cancer.

**Fig. 4:**
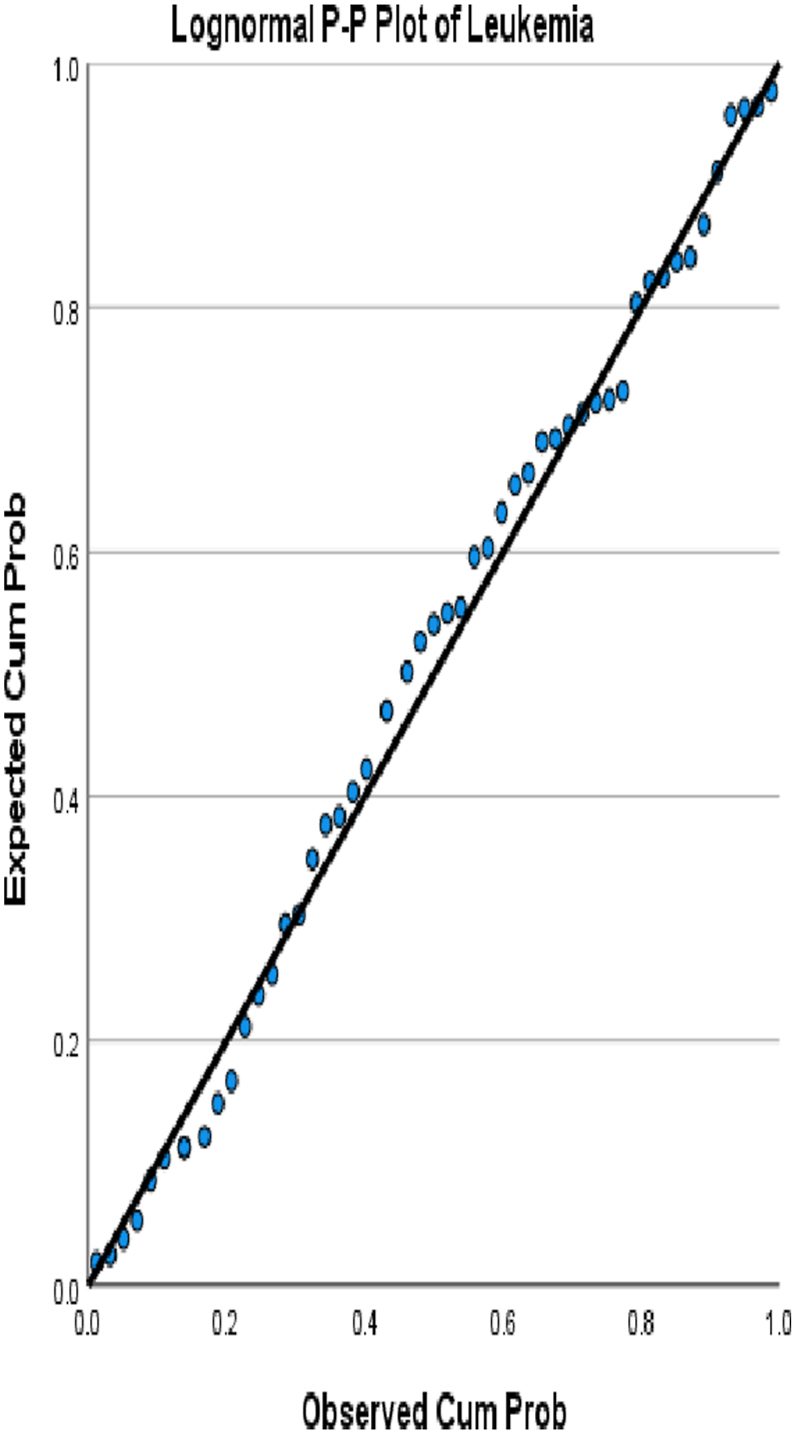
Leukemia cancer.

**Fig. 5:**
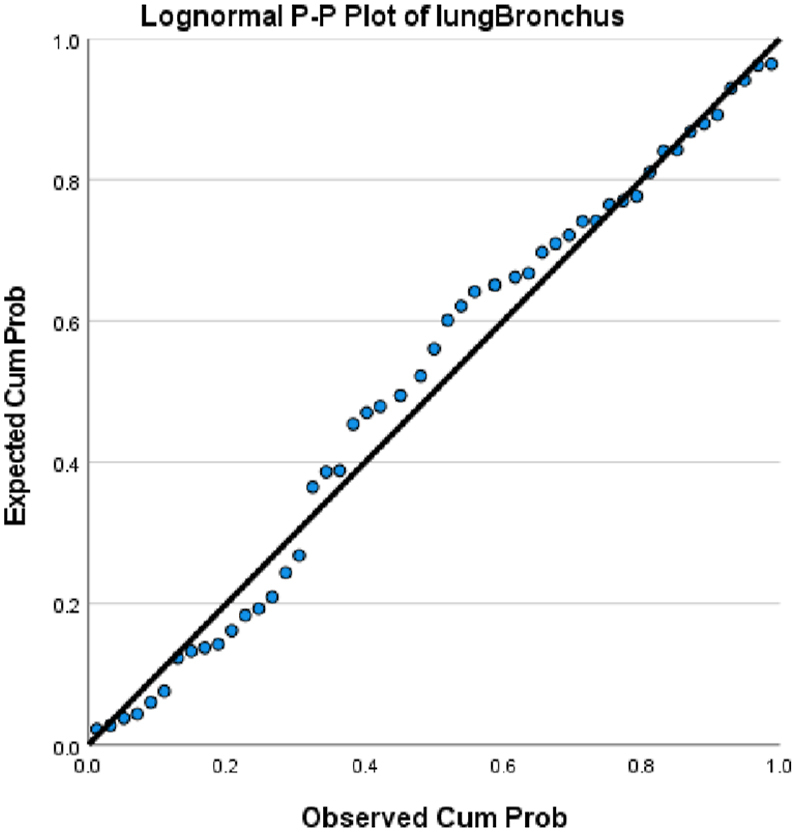
Lung cancer.

**Fig. 6:**
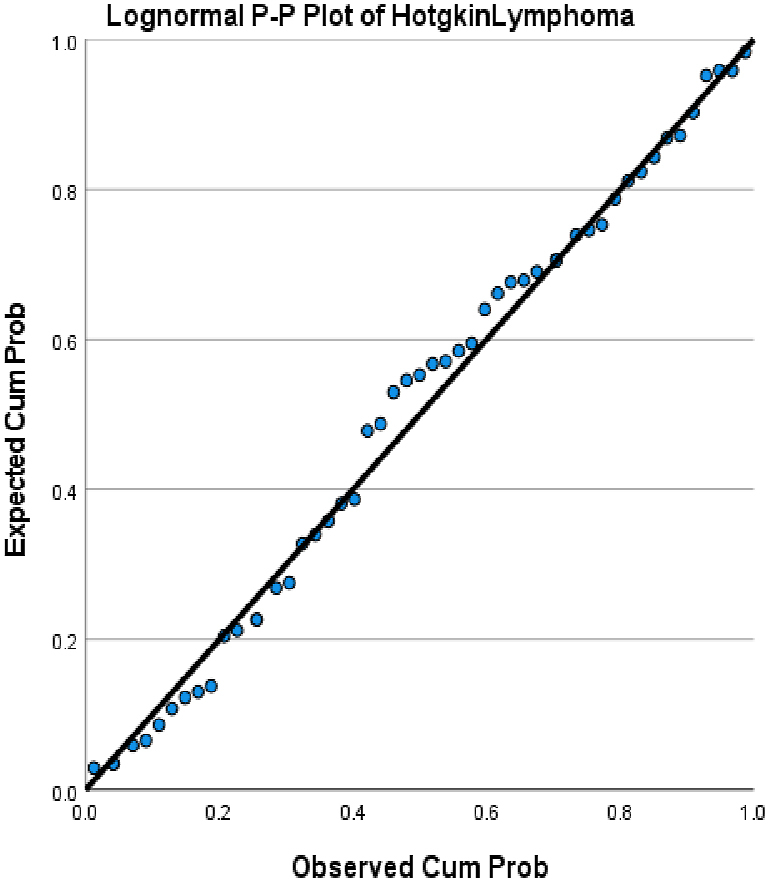
Lymphoma cancer.

**Fig. 7:**
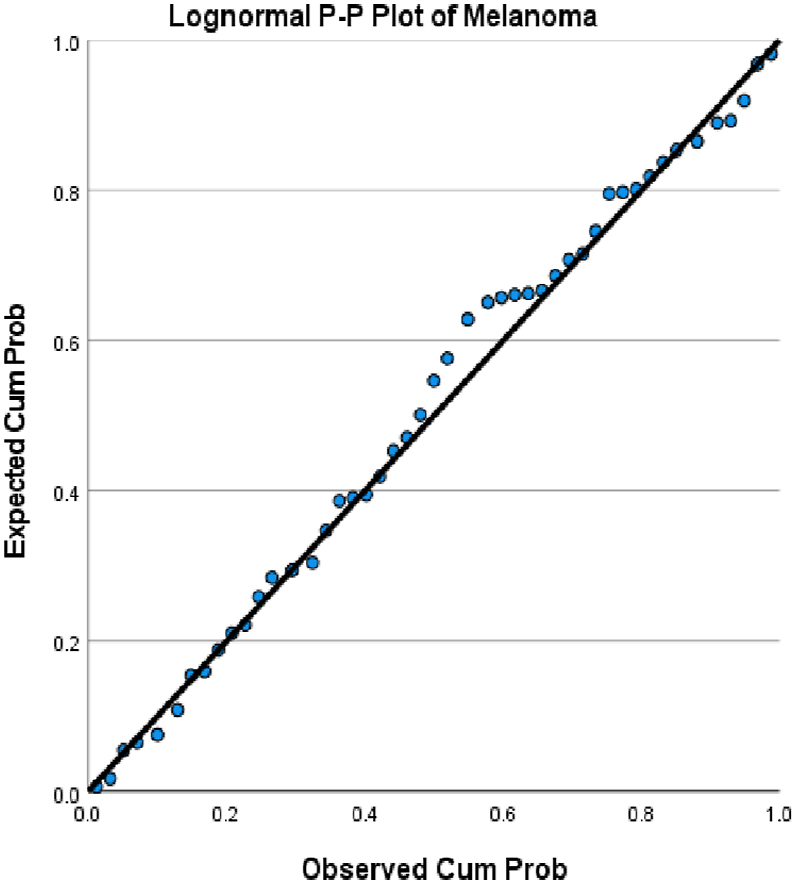
Melanoma cancer.

**Fig. 8:**
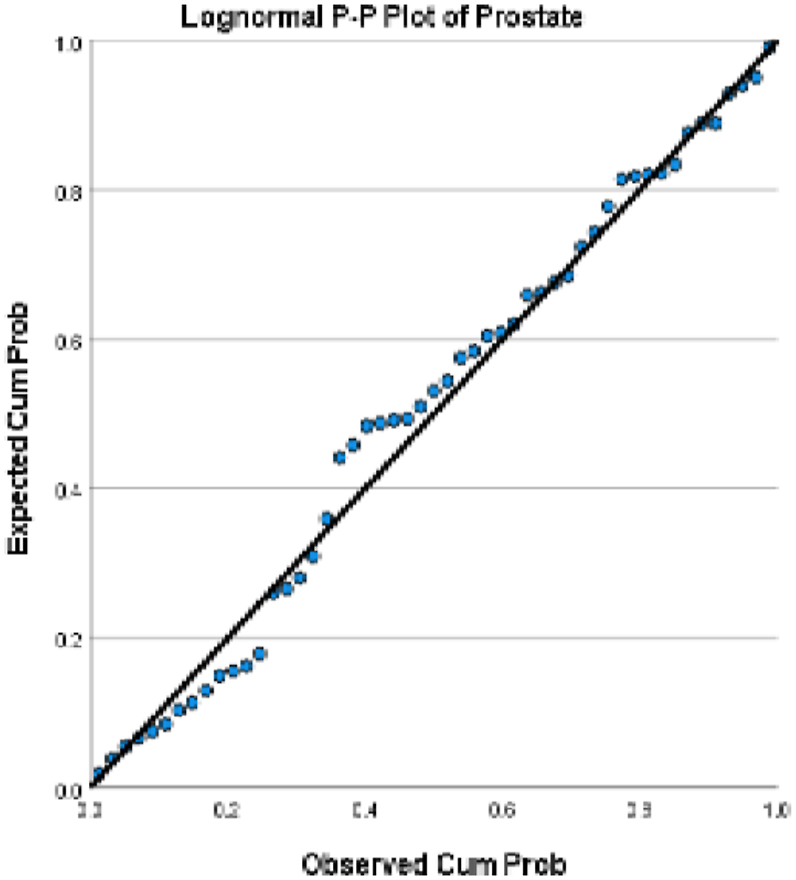
Prostate cancer.

**Fig. 9:**
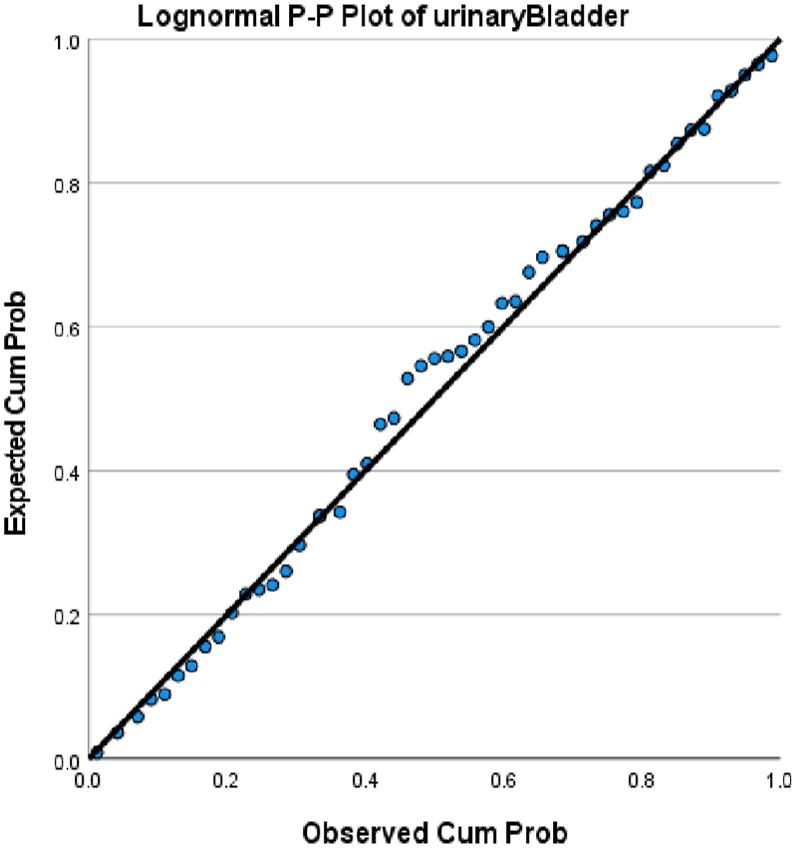
Urinary cancer.

**Fig. 10:**
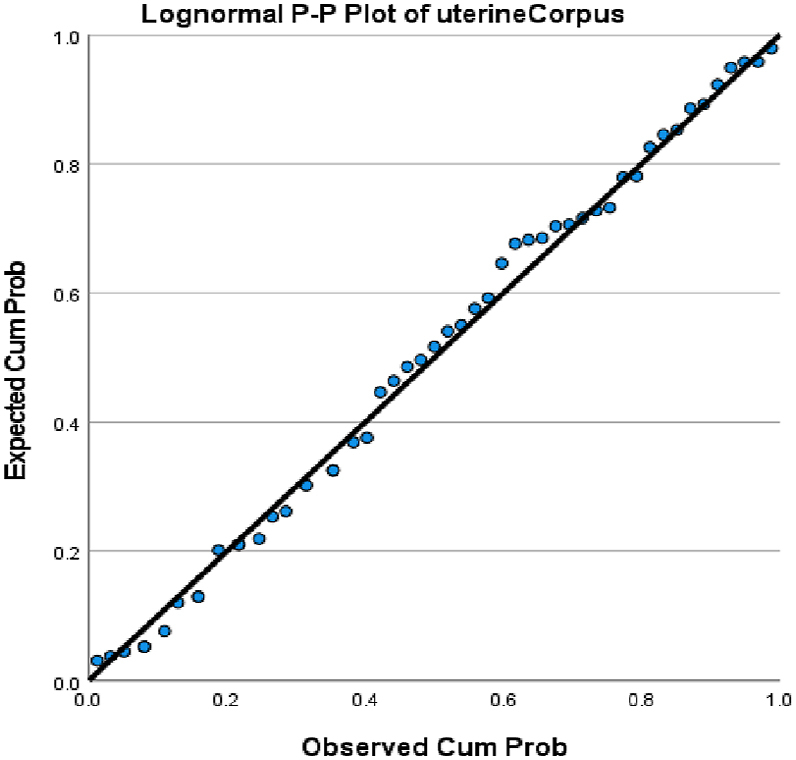
Uterine corpus cancer.

We choose in this article to study the top ten cancers, because they have the highest incidence rates. They are breast, cervical, colorectal, lymphoma, leukemia, lung/bronchus, melanoma, prostate, urinary, and uterine/corpus cancers. These cancer incidences are grouped in terms of three epochs: pre-COVID-19 (2017, 2018), during COVID-19 (2019, 2020, 2021), and post-COVID-19 (2022, 2023) using a data analytic approach. The incidences of these ten cancers tend to not be Gaussian distributed. The Gaussian distributional data is often a requirement for popular statistical analysis. After more scrutiny of other stochastic patterns like the Poisson, inverse binomial, lognormal for the cancer incidences, we noticed that the incidences of each of the ten cancers mentioned above closely follow a lognormal stochastic pattern. This can be observed in the Probability (PP) plots in Fig. 1, Fig. 2, Fig. 3, Fig. 4, Fig. 5, Fig. 6, Fig. 7, Fig. 8, Fig. 9, and Fig. 10.

The tightness of the x-y points to the positive diagonal line in the P–P plot provides evidence that the underlying stochastic pattern of the data is the lognormal type. See Shanmugam and Chattamvelli [13] for details on lognormal stochastic patterns. The lognormal P–P data plots of breast, cervical, colorectal, leukemia, lung & Bronchus, lymphoma, melanoma, prostate cancers, urinary, and uterine corpus are displayed above.

On March 11, 2020, the World Health Organization (WHO) declared that COVID-19 was a global pandemic. Almost immediately, normal day-to-day activities were impacted, and this was especially true in the health care industry. Health services, including cancer diagnostic tests and treatment of noncommunicable diseases, were delayed due to COVID-19. Cancer and noncommunicable diseases were given a low priority in addition to delaying and canceling elevated risk cases, which adversely affected immunocompromised cancer patients. This is not without reason. Thus, preventive medical efforts such as screening, extra isolation strategies, specialized vaccinations, or pertinent systemic therapies normally made available to cancer patients were curtailed based on competing risk to allow hospitals and health systems to effectively combat the surge of COVID-19 cases [14,[15], [16], [17]]. As a result, a review of the impact of COVID-19 on diagnosis and treatment of cancers revealed that there was a 27% decrease in diagnosis of cancers throughout the pandemic [18]. In just one example, the COVID-19 pandemic devastated the Catalan population in Spain and forced both public and private hospitals to cease treating cancer patients [19]. Prior researchers discovered that patients with cancer were at a higher risk of COVID-19 complications and even death compared with non-cancer COVID-19 patients [20]. The clinical outcomes in patients with cancer and COVID-19 were complicated. The cytokine storm due to COVID-19 caused high morbidity and mortality. The association between COVID-19 and the incidence of cancers was also examined in the province of Manitoba, Canada [21]. The findings were that there was an initial decrease in cancer diagnosis and consequently, a high cancer fatality was noticed. Hospital admissions for the treatment of lung cancer were reduced significantly during COVID-19 [22]. With an interest to measure the association between patient-reported disruptions of service and healthcare services to cancer patients, a study revealed that one out of every three cancer patients experienced a disruption of service with a higher rate of anxiety [22]. Several studies reported a major impact on melanoma cancer due to diminished screening because of COVID-19. The chronic obstructive pulmonary disease was more severe among patients diagnosed with COVID-19 compared with patients who were not diagnosed with COVID-19 [23].

COVID-19 interrupted healthcare services. The absence of early detection, sickness matriculated into cancer occurrence, and it might have triggered the cancer incidence rate due to the pandemic. This might have happened with respect to several cancers including melanoma and other skin cancers. There also were challenges to the medical community as some of the melanoma cancer patients had to be admitted to the emergency wing for treatment during COVID-19 era. Admissions to the hospital for dementia treatment also increased significantly during COVID-19. More severe lung and bronchus cancer cases were admitted in respiratory clinics [24]. The COVID-19 pandemic impacted the pattern of treating cancer patients, in-hospital mortality, and the number of patients joining intensive care unit of the hospital due to COVID-19, irrespective of whether the patients were vaccinated or not [25]. The American Cancer Society warned that the new cancer cases had increased due to COVID-19. Recall that the estimated incubation time of COVID-19 is crucial to return to the normal operation style regarding treating cancer patients and it is about (exact number is yet unavailable) fourteen days per case. Until COVID-19 is under control, the delay in treating cancer patients is not going to be eliminated [26]. Medical professionals have speculated that the presence of COVID-19 virus might damage the healthy functioning of the organs which might cause cancer cells eventually [27]. The average time to surgery since the patient's referral was delayed by 119 days (about 4 months) for head, neck, and skin cancers patients due to COVID-19 [27]. The U.S. veteran's community is no exception to the impact of COVID-19. In fact, a general linear modeling of U.S. veteran's data revealed that the comorbid conditions were more prevalent among veterans than among non-veterans, during pre as much as post-COVID-19 eras [28]. At least for cancer patients, preventive medical efforts such as screening, extra isolation strategies, specialized vaccinations, or pertinent systemic therapies ought to be made available [[29], [30]]. The lockdown in response to COVID-19 in United Kingdom caused the suspending of cancer screening and it impacted an increase of breast and lung cancer. Cancer patients' immune system is increased by appropriate immunotherapy. The COVID-19 has created stress on healthcare providers worldwide [31].

A literature review of the impact of COVID-19 on cancer is worthwhile to realize the importance of this research topic before we proceed to outline the research goal of our research in this section. During COVID-19, income for some employees was less, some lost job, some lost insurance coverage, and had a decreased access to hospital/medical provider. These negatively impacted them with less ability to obtain timely screening or diagnosis for breast cancer, according to Ref. [32]. Murthy et al. [33] reported that the COVID-19 significantly delayed cancer surgery. Schafer et al. [34] pointed out that the COVID-19 disrupted health care and a decline in almost all cancer diagnosis. Pini et al. [35] described the impact of COVID-19 on mental health of people living with breast, prostate, and colorectal cancers. Fritsch et al. [36] reported that COVID-19 decreased the number of screenings for mammography. Ribeiro et al. [37] disclosed that COVID-19 had a significant impact on breast cancer patients. Teresa Ardillo et al. [38] mentioned a confusion due to influenza vaccines versus COVID-19 mRNA vaccines might have caused more fear among cancer patients than the public. Baek et al. [39] narrated that COVID-19 caused an increase in the number of cardiovascular patients to the emergency department in Korea. Gupta et al. [40] hypothesized that COVID-19 was connected to a decreased cancer physiatry referrals in 2020 and confirmed its validity with data. Kasputis et al. [41] declared that COVID-19 continued to affect the behavior of cancer patients in their home environments even after the termination of the pandemic due to the fear of infection versus vaccination. After defining financial toxicity, Wu et al. [42] pronounced that cancer patients underwent financial distress which adversely affected clinical outcomes. Using cancer data during 2015–2020, Serban Negoita [43] evaluated the impact of the COVID‐19 pandemic on cancer incidence counts, using regression and ratios of the observed‐to‐expected number of cancers. Among the six cancer types: colorectal, female breast, lung, pancreas, prostate, and thyroid, Negoita noticed that the impact of COVID‐19 on prostate cancer was higher among Black persons in the U.S. However, the literature review implies that there is a dearth of research assessing the impact of COVID-19 on cancer incidence. Our research has been to explore the impact of the pandemic COVID-19 on the incidences of cancer in the US. To address the research gap, the aims of this paper are conceived as discussed in the subsection below. The aim of this study is to assess the impact of COVID-19 on cancer incidence with a special focus on the top-10 cancer sites in the US. In this research process, we theorized that the pandemic changed reporting and treatment of cancer. The latter was due to the delay in treatment and could also be caused by a mutation of cancer itself from the COVID-19 virus. This is the novelty of our work. Our findings are not congruent with previous literature, and we believe it is entirely due to the pandemic.

The observed cancer incidences are correlated. That is, the vectors of cancer incidences in terms of years are non-orthogonal in a Euclidean space. The principal component analysis (PCA) is a multivariate technique which orthogonalizes correlated vectors. The values in the PCA are the basis on which the contents of the manuscript have been built and this approach is a novelty since no one has tried this way.

Given that the literature has pointed out the evidence of many effects of COVID-19 on cancer, this article confirms that the incidences shifted within pre, during, and post-COVID-19 eras. Furthermore, a significant turn in the incidences occurred in the peak year 2020 of the COVID-19. We have calculated the (exceedance) probability of each among the ten cancers to have more than 1000 cases in a future year. For this confirmation, we have considered a novel and innovative (nontraditional) approach using the first two principal components of a multivariate data reduction technique, the minimum-maximum transformation of the lognormally distributed ten major cancer incidences.

Partially in response to COVID-19, there has been a resurgence of interest in the scholarly literature on fortifying networks to safeguard against sudden disruptions including the spread of epidemics. Much of this literature draws from percolation theory, where nodes (individual points) and edges (connection between points) are randomly removed or immunized in a large network to analyze its resilience. However, in real world settings, especially rapidly developing pandemics, the knowledge of which nodes to remove or immunize to protect the network may be limited. Liu et al. [44] have developed a targeted immunization framework where only *n* nodes may be visible at a time, and the most central of those *n* nodes are immunized. They conclude even for a small *n*, this approach yields significant benefits. Shang [45] has extended their work by accommodating not only limitations in space (knowledge of nodes) but also limitations in time as the node immunity may be temporary in nature. His results suggest that increasing the level of knowledge in targeted immunizations may not yield the anticipated effectiveness in combating certain outbreaks, such as COVID-19.

We now present additional details about the utilized dataset in this study. All we know (from the source https://www.cancer.org/research/cancer-facts-statistics/all-cancer-facts-figures) is the number of cancer cases in each one of the US States. The data do not explicitly reveal whether the COVID-19 had an impact on the cancer incidences. Recognizing that COVID-19 was noticed in year 2019 but prolonged up to year 2021, we decided to consider three epochs: years 2017, 2018 as the pre-COVID, years 2019, 2020, and 2021 as during COVID time, and years 2022, 2023 as post-COVID to compare the cancer incidences. To compare the three pre-, during-, and post- COVID periods, we encountered a technical difficulty that the vectors of cancer incidences happened to be correlated. That is, the vectors of cancer incidences in terms of years are non-orthogonal in a Euclidean space. The principal component analysis (PCA) is a multivariate technique which orthogonalizes correlated vectors. The values in the PCA are the basis on which the contents of the manuscript have been built and this approach is a novelty since no one has tried this way. Consequently, results from the PCAs helped to compute the angles between the cancer incidence vectors, to notice that the cancer incidences shifted within each era (pre, during, and post), with a meaningful change in the cancer incidences occurring in 2020, the peak of the COVID-19 era, and to interpret the exceedance probability for a cancer type to have 1000 incidences in a future year among the breast, cervical, colorectal, uterine corpus, leukemia, lung & bronchus, melanoma, Hodgkin's lymphoma, prostate, and urinary cancers.

## 2 Materials and methods

The principal components analysis (PCA) calculates a change in direction of principal components. Our research focus was on whether the major cancer incidences have shifted during the periods (1) pre-COVID-19 era, (2) COVID-19-time era, and (3) post-COVID-19 era. To answer the shifts with respect to cancer type and with respect to the three eras, PCA is a useful data analytic tool. Their shifts are not directly observed but only their incidences are observed. The PCA is helpful to notice the shifts. By sketching the relative proximity of the ten cancers in a two-dimensional Euclidean graph (with the first principal component value in horizontal axis and the second principal component value in the vertical axis), the angles between the adjacent cancer incidence vectors for each year show direction of incidence for a pair of cancers. Using PCA to map multiple years’ incidence shows change in direction over time. These angles became the basis for tracking not only the dynamics and volatility of each cancer in the pre, during and post eras of COVID-19, but it also helped to create and interpret unique indices for each cancer incidence within as well as across the three eras. No article in the literature has considered our approach which provides a clear insight of the effects of COVID-19 on the ten cancer incidences in the pre, during, and post eras.

The angles for all cancers and all years were calculated and charted. We calculated and charted the angles of depression for all pairs of adjacent cancers. We also included a radar plot of these angles. To further understand the results, we calculated and charted hazard rates.

## 3 Data analytic results of US cancer data in pre, during, and post-COVID-19 eras

The cancer incidences, denoted by the random variable **X** with element x, follow most closely a lognormal stochastic pattern. The probability for x to be more than t in the interval (0,∞) is $S\left(t\right)=\mathrm{Pr}\left[x\geq t\right]=\bar{\Phi }\left(\frac{\ln\;t-\hat{\mu }}{\hat{\sigma }}\right)$,where $\bar{\Phi }\left(m\right)=\int_{m}^{\infty }e^{-t^{2}/2}dt/\sqrt{2\pi }$ is the standardized Gaussian cumulative function with m representing the typical location and scale transformation with 0<μ<∞ and σ>0 are location and shape parameters respectively. The sample data are indicated by $\left(x_{1},x_{2},\ldots x_{n}\right)$. The estimate of the location and shape parameters are ${\hat{\sigma }}^{2}=\ln\left(1+\frac{s_{x}^{2}}{\bar{x}}\right)$ and $\hat{\mu }=\ln\left(\bar{x}\right)-\frac{1}{2}\ln\left(1+\frac{s_{x}^{2}}{\bar{x}}\right)$ where $s_{x}^{2}$ and $\bar{x}$ are the data dispersion and average respectively are variance and average respectively. The hazard rate for cancer incidence, $\hat{h}\left(t\right)=\frac{1}{t\hat{S}\left(t\right){\hat{\sigma }}_{t}}\varphi \left(\frac{\ln\;t-\hat{\mu }}{\hat{\sigma }}\right)$ where $\varphi \left(z\right)=\frac{1}{\sqrt{2\pi }}e^{-z^{2}/2}$ is the Gaussian probability density function [31]. The hazard rate is indicative of how many more cases of a particular type could occur in the year if a person has survived to that year.

The most likely cancer incidence (that is, the mode) of the lognormal stochastic pattern occurs at its unimodal, ${\hat{M}}_{mode}=\bar{x}{\left(\frac{\bar{x}}{\bar{x}+s_{x}^{2}}\right)}^{1.5}$. Hence, we wish to consider ${\hat{M}}_{mode}$ as an index to compare the status of each cancer incidence over the eras before, during, and after the COVID-19. Because the cancer incidences, data average and dispersions are extremely large, it is a necessity to consider a uniform linear transformation (also known as min-max scaling), $u=\frac{x-x_{\min}}{x_{\max}-x_{\min}}$ of the data so that the new numbers are standardized to be in the interval [0, 1], before constructing principal components (PC) to capture the proximity among the cancers during any one of the three eras of time. In the transformed data, note that $u_{mode}=\frac{u-u_{min}}{u_{\max}-u_{\min}}$, where the notations $u_{\min}$, and $u_{\min}$ denote respectively the minimum and maximum in each cancer data $\left(u_{1},u_{2},\ldots ,u_{n}\right)$ of sample size n=51 of the US states including District of Columbia (DC). The unimodality of the transformed uniform values occurs at ${\hat{U}}_{\mathrm{mod}\;e}=\bar{u}{\left(\frac{\bar{u}}{\bar{u}+s_{u}^{2}}\right)}^{1.5}$. By reversing the translation, we notice that ${\hat{M}}_{mode}=x_{\min}+\left(x_{\max}-x_{\min}\right){\hat{u}}_{\mathrm{mod}\;e}$, meaning that the most likely cancer incidence is the minimum incidence plus the range times the mode, n $\left(x_{\max}-x_{\min}\right){\hat{u}}_{\mathrm{mod}\;e}$ in which the range $\left(x_{\max}-x_{\min}\right)$ amplifies the ${\hat{u}}_{\mathrm{mod}\;e}$ in the interval [0, 1]. One should note that the larger range refers to a heterogeneous performance while the smaller range portrays more homogeneous performance on a cancer incidence by absence, presence, or disappearance of COVID-19. The range is another index to educate us the level of a cancer's connection to COVID-19, although each is susceptible to outliers.

To exercise the full power of PCA, we needed to calculate each PC. We used the software IBM SPSS – 28 to obtain the 1st and 2nd PC scores to construct the two-dimensional proximity structures of the 10 cancer incidences. The entry in the data matrix is the number of incidences in U. S. with a cancer type in a row and a year in a column. There are ten rows for cancer types and eight columns for the years starting in 2017 and ending in 2023. With the PCA results and the basic trigonometric concepts, we have quantified the cosine angle of inclination of cancers with each other in a novel manner and then utilized the angles to create indices to rank the severity in their incidences during the three eras (before, during, and after) the COVID-19. Interestingly, we document a nontrivial shift in the cancer proximities in the raw data of incidences. Our data analytic approach and interpretations of the results do follow up after the literature review in our research direction.

**Fig. 11:**
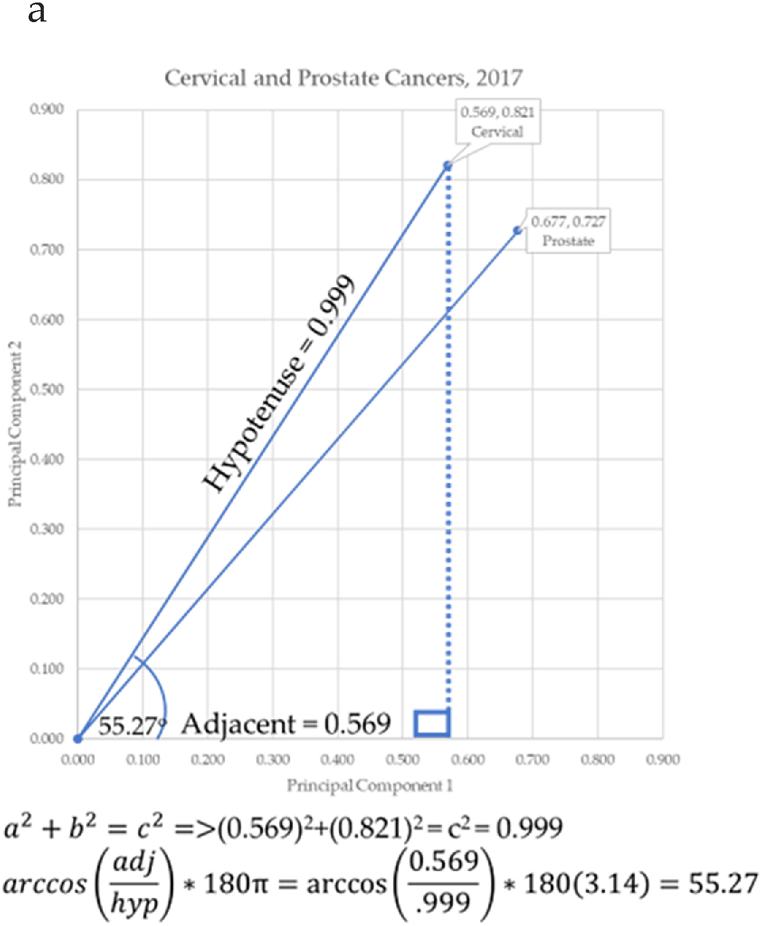

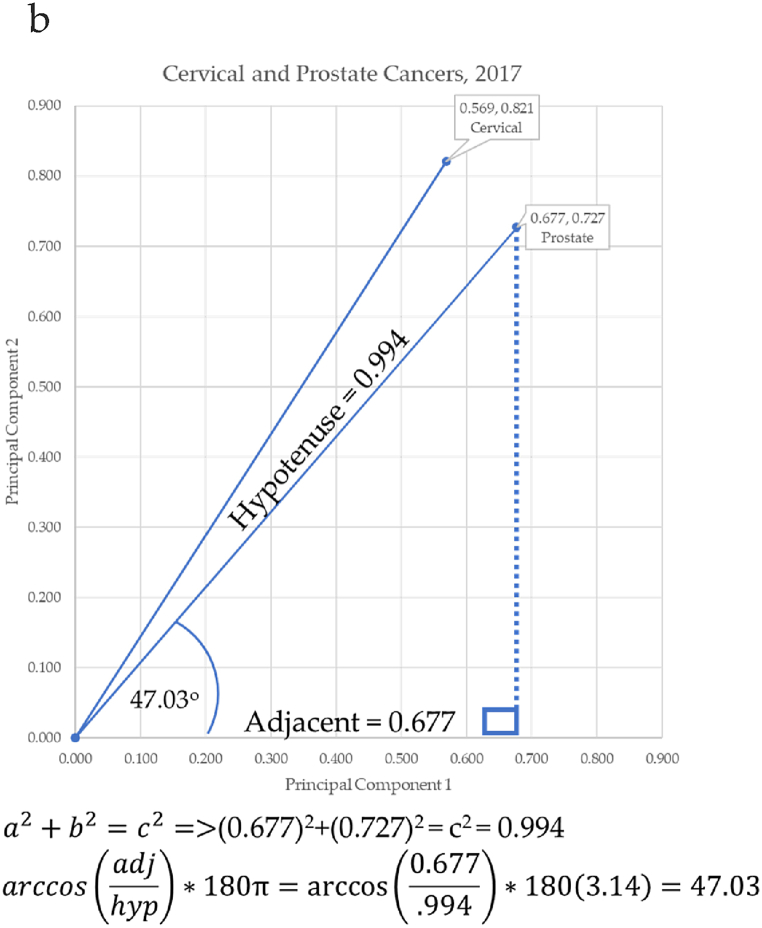

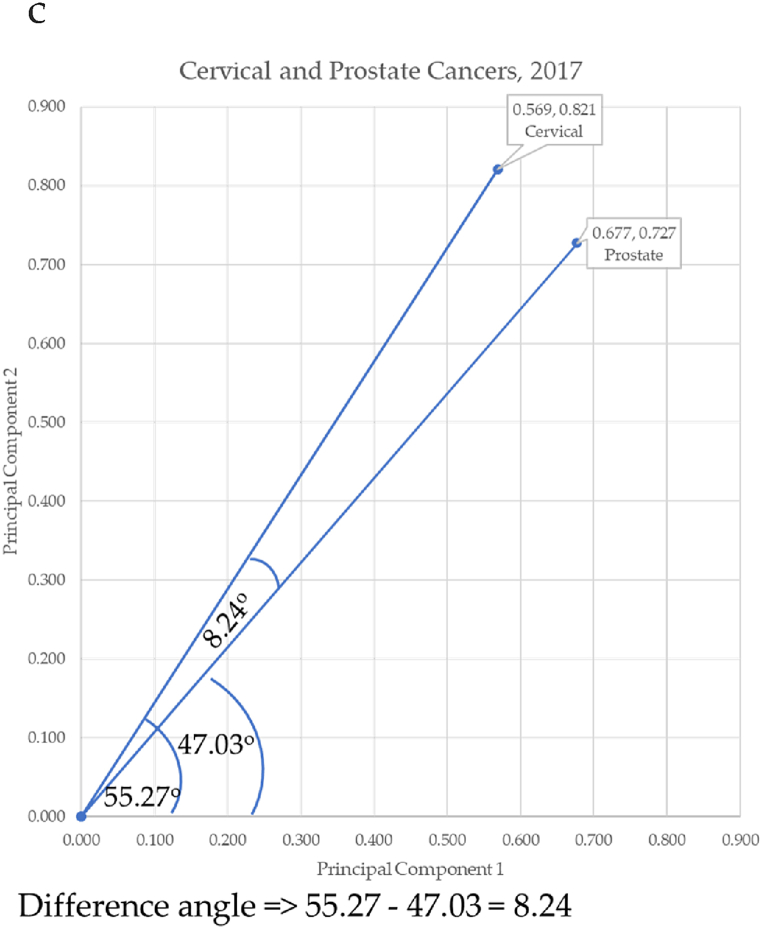

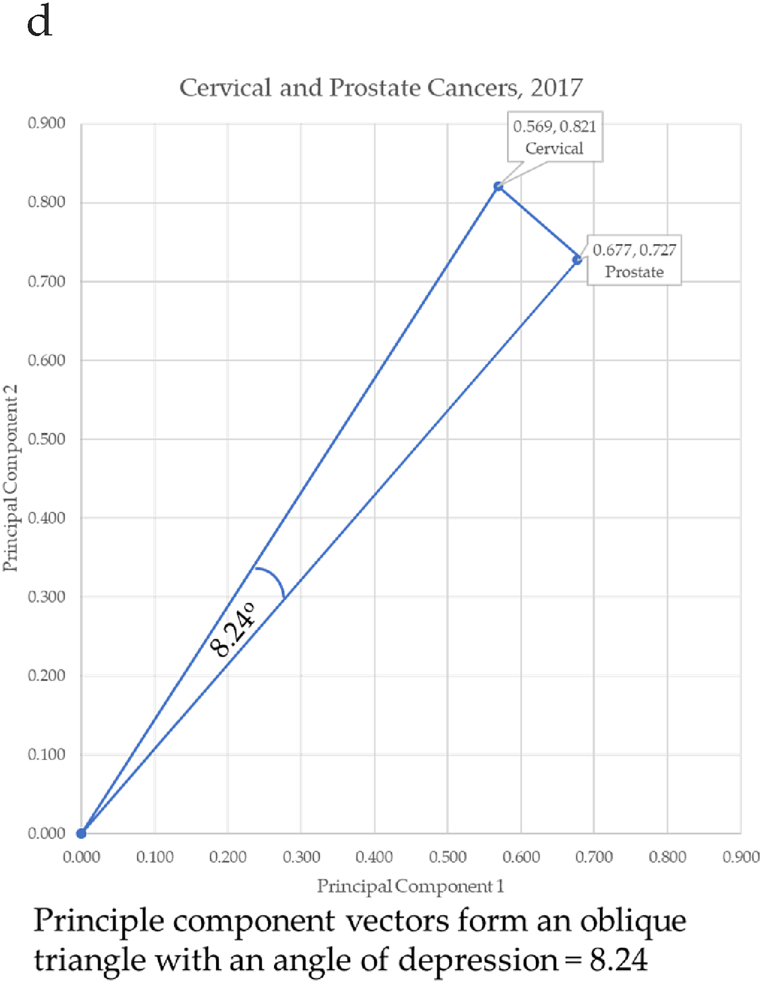
a. Angle to cervical cancer, b. Angle to prostate cancer, c. Calculating the angle to cervical and prostate cancer, d. Angle between cervical and prostate cancers

Principal components were used as Cartesian coordinates to form vectors for 10 forms of cancer: breast, cervical, colorectal, leukemia, lung & Bronchus, lymphoma, melanoma, prostate cancers, urinary, and uterine corpus. These cancers were charted over seven years: 2017–2023. Three eras are considered. The first is pre-COVID-19 (2017–2019), second era is during-COVID-19 (2019–2021), and the post-era of COVID-19 is (2022–2023). Calculation of the angle differences between cancer vectors was performed through vertical drop lines in Excel. This enabled the calculation of the length of the vector (the hypotenuse) through the Pythagorean Theorem, and the calculation of the angle of depression through the arccosine (ACOS) function as shown in Fig. 11a through Fig. 11d below. ACOS was converted to degrees by multiplying by 180*π.

Fig. 11a–d shows the progressive calculation of the angle of depression between cervical and prostate cancers. This same process was used for all cancer pairs of adjacent cancers.

**Fig. 12:**
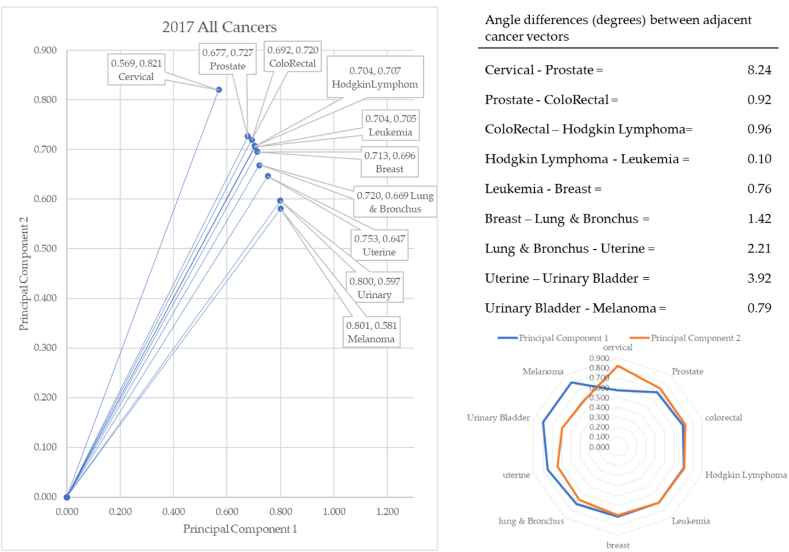
Contiguity among cancers in 2017.

Fig. 12 shows the proximity among the cancers. In 2017, cervical cancer formed the vector with the most extreme angle (55.27°) and melanoma with the most conservative angle (36.96°). Between cancer vectors, the widest angle of depression was between cervical and prostate cancer (8.24°). The smallest angle of depression was between Hodgkin Lymphoma and leukemia (0.10°). The total angle vectors for 2017 summed to 19.31°. The angles defined a difference between incidence of each cancer. The pre-pandemic years of 2017–2019 created a baseline from which to compare angles during and post pandemic. The data will demonstrate that the incidences of cancers have shifted due to the pandemic.

In 2018, cervical cancer formed the vector with the most extreme angle (55.29°) and melanoma with the most conservative angle (38.05°): consistent with 2017. Between cancer vectors, the widest angle of depression was between cervical and prostate cancer (8.74°) and the smallest angle of depression was between Hodgkin Lymphoma and lung & Bronchus (0.20°): similar to 2017. The total angle vectors for 2018 summed to 20.58°. This was 7% greater than 2017.

**Fig. 13:**
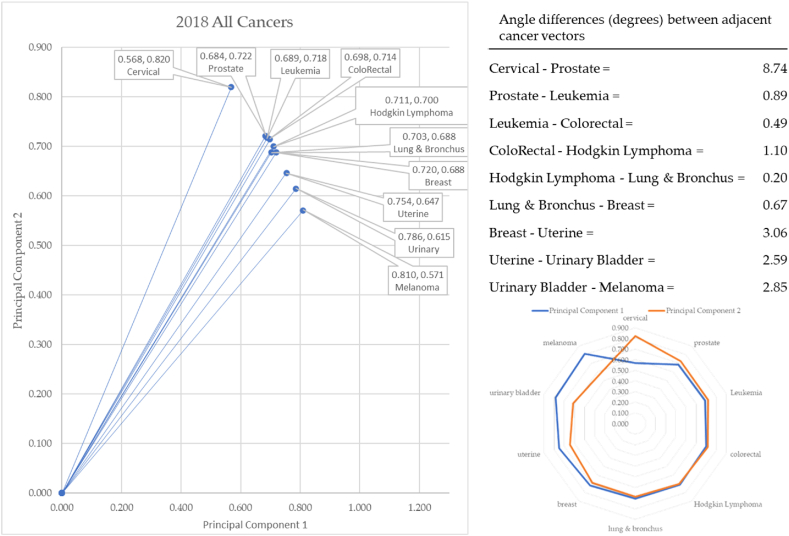
Contiguity among cancers in 2018.

The year 2018 served as the second year in our baseline data. There were small angle differences between these years, but nothing material (see Fig. 13). For instance, the angle between cervical and prostate cancer changed from 8.24 to 8.74. The five tenths of a change on principal component data was not material. In 2018, the order of cancer angles changed slightly, but this was also not a material difference. For instance, in 2017 the second angle was between prostate and colorectal, but in 2018 the leukemia vector increased its angle of depression from 45.05 to 46.16. This brought it up next to the prostate cancer vector.

**Fig. 14:**
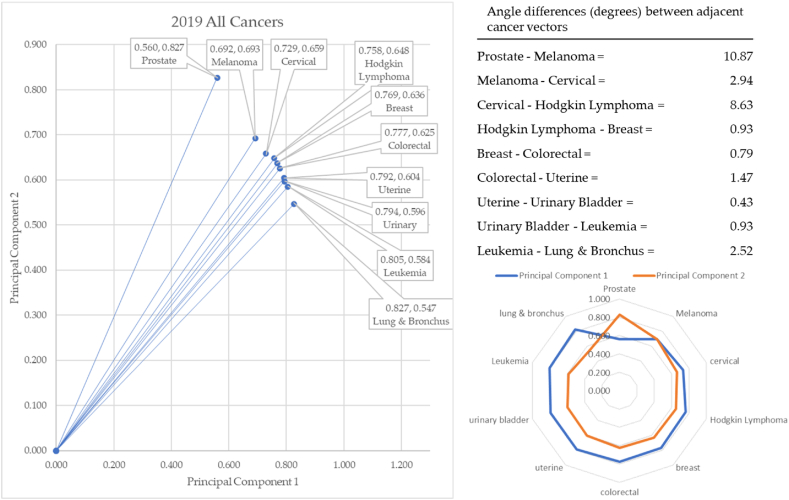
Contiguity among cancers in 2019.

In 2019, prostate cancer formed the vector with the most extreme angle of depression (55.9°) and lung & bronchus with the most conservative angle (33.47°), as illustrated in Fig. 14. Between cancer vectors, the widest angle of depression was between prostate and melanoma (10.87°) and the smallest angle of depression was between uterine and urinary cancer (0.43°). The total angle vectors for 2018 summed to 20.58°. The total angle vectors for 2019 summed to 29.51°. This was 43% greater than 2018 and 53% greater than 2017.

The year 2019 served as the last year of our baseline data (pre-pandemic). Prostate cancer replaced cervical cancer as the cancer with the highest angle of depression. Melanoma rose from cancer with the smallest angle of depression to the second highest. The total change in angle was 10.09° in 2018 and 9.34° from 2017. A reason for writing this manuscript is that we theorized that the pandemic changed reporting and treatment of cancer. The latter was due to the delay in treatment and could also be caused by a mutation of cancer itself from the COVID-19 virus. This is the novelty of our work. Our findings are not congruent with previous literature, and we believe it is entirely due to the pandemic.

**Fig. 15:**
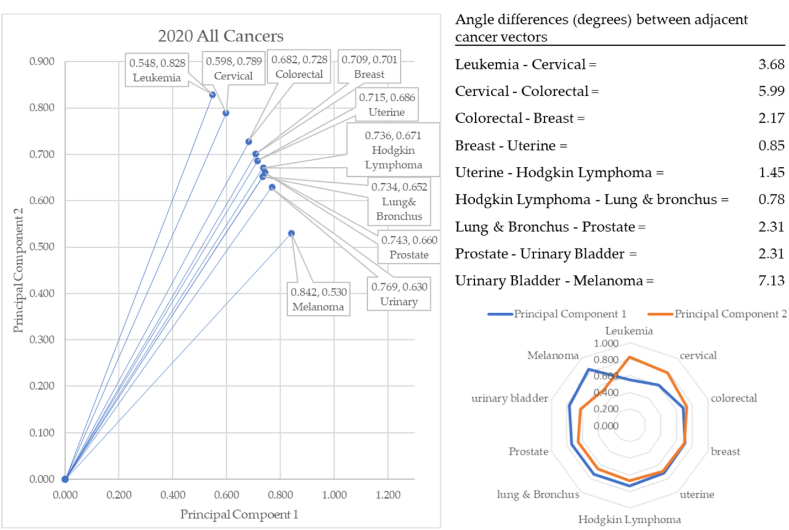
Contiguity among cancers in 2020.

Fig. 15 shows how in 2020, leukemia formed the vector with the most extreme angle (56.51°) and melanoma with the most conservative angle (32.18°). Between cancer vectors, the widest angle of depression was between cervical and colorectal (5.99°), and the smallest angle of depression was between Hodgkin Lymphoma and lung & bronchus (0.78°). The total angle vectors for 2020 summed to 26.66°. This was 38% greater than 2017, 30% greater than 2018, and 10% less than 2019.

This served as the first of two years of pandemic data. Leukemia and cervical cancer rose to the top of this set of data. Melanoma's angle of depression returned to pre-pandemic levels (32.18°). This is 3.03 and 3.78° less than 2018 and 2017, respectively.

**Fig. 16:**
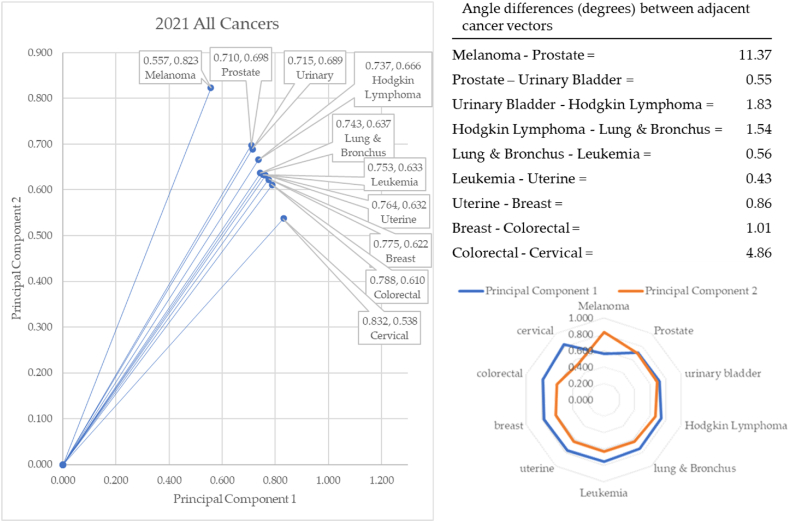
Contiguity among cancers in 2021.

Fig. 16 illustrates that for 2021, melanoma formed the vector with the most extreme angle (55.88°) and cervical cancer with the most conservative angle (32.88°). Between cancer vectors, the widest angle of depression was between melanoma and prostate cancer (11.37°) and the smallest angle of depression was between leukemia and uterine cancer (0.43°). The total angle vectors for 2021 summed to 23.00°. This was 19% greater than 2017, 12% greater than 2018, 22% less than 2019, and 14% less than 2020.

In 2021, melanoma rose to the top again, like in 2019. Cervical cancer's angle of depression dropped to 32.88°, which is unlike any other year in the baseline data. The bottom three angles of depression (cervical, colorectal, and breast) are all cancers for which screening is available and highly effective.

**Fig. 17:**
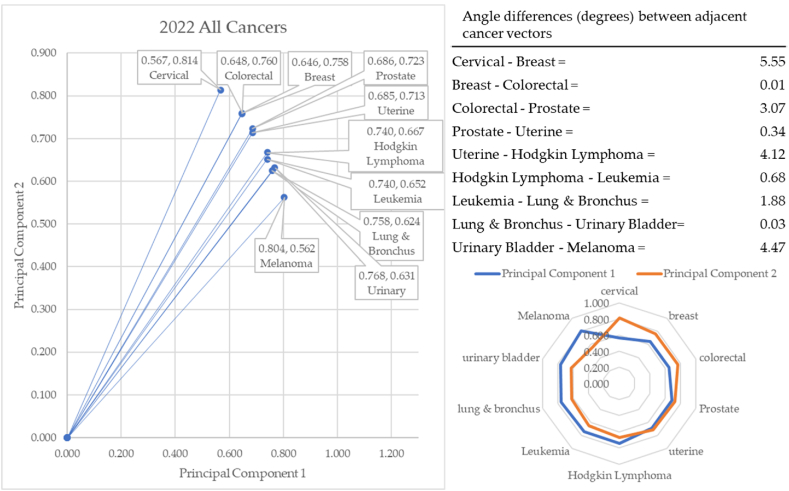
Contiguity among cancers in 2022.

In 2022, cervical cancer formed the vector with the most extreme angle (55.12°) and cervical cancer with the most conservative angle (34.97°), as depicted in Fig. 17. Between cancer vectors, the widest angle of depression was between cervical and breast cancer (5.55°) and the smallest angle of depression was between lung & bronchus and urinary cancer (0.03°). The total angle vectors for 2022 summed to 20.15°. This was 4% greater than 2017, 2% less than 2018, and 32% less than 2019, 24% less than 2020, and 12% less than 2021.

Like the years 2017 and 2018 (pre-pandemic), cervical cancer rose to the top of cancer angles of depression. The top four angles were cervical, breast, colorectal, and prostate. These are cancers for which screening is readily available and highly effective.

**Fig. 18:**
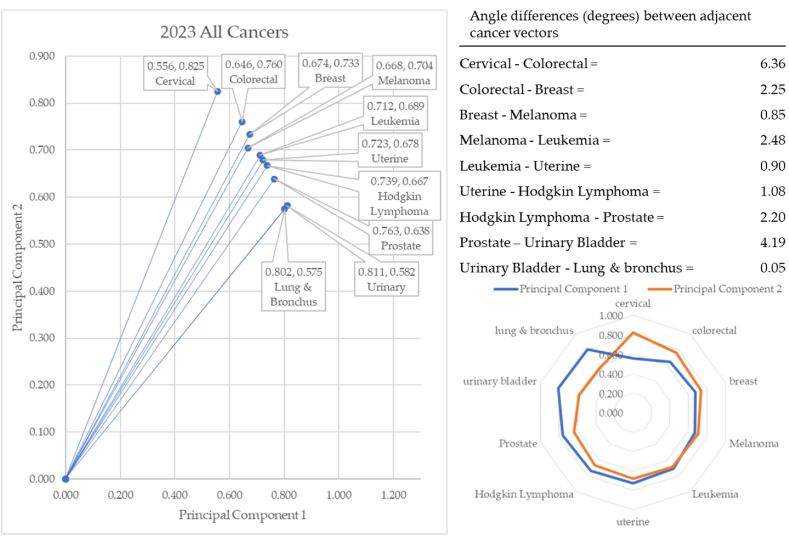
Contiguity among cancers in 2023.

**Fig. 19:**
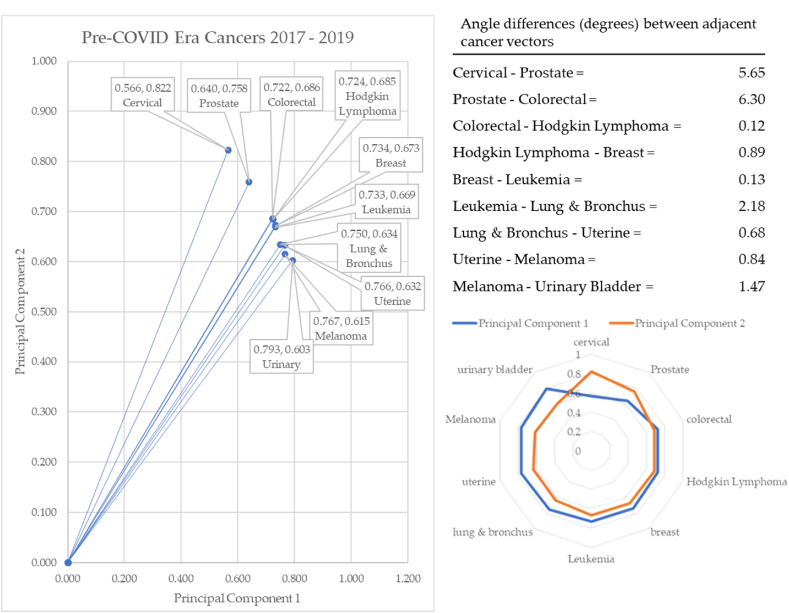
Dispositions in pre-COVID era (2017–2019).

In 2023 (see Fig. 18), cervical cancer formed the vector with the most extreme angle (55.99°) and lung & bronchus cancer with the most conservative angle (35.64°). Between cancer vectors, the widest angle of depression was between cervical and colorectal cancer (6.36°) and the smallest angle of depression was between urinary and lung & bronchus (0.05°). The total angle vectors for 2023 summed to 20.36°. This was 5% greater than 2017, 1% less than 2018, and 31% less than 2019, 24% less than 2020, 12% less than 2021, and 1% greater than 2022. The dispositions of the angles in pre-COVID era (2017–2019) are shown in Fig. 19.

The year 2023 was the final year in which we collected and processed data. This formed the second year of the post-pandemic group. We would expect things to return to pre-pandemic conditions, but several cancers exhibit different incidences than prior to COVID-19. We chose to group the pre, during, and post pandemic eras for analysis.

**Fig. 20:**
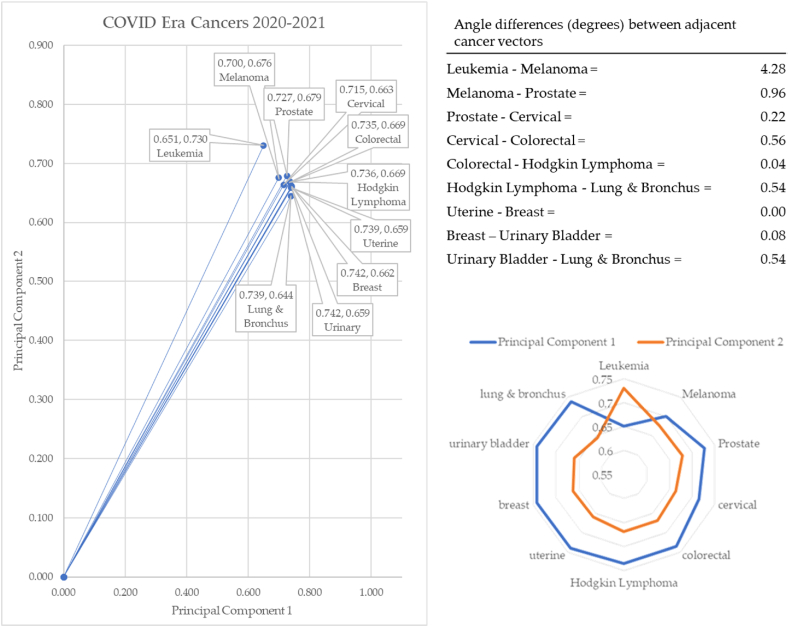
Dispositions in during COVID era (2019–2021).

In the pre-COVID-19 years of 2017–2019 (shown in Fig. 19), cervical cancer formed the vector with the most extreme angle (55.49°) and urinary cancer with the most conservative angle (37.24°). Between cancer vectors, the widest angle of depression was between prostate and colorectal cancer (6.30°) and the smallest angle of depression was between colorectal and Hodgkin Lymphoma (0.12°). The total angle vectors for the pre-COVID-19 era summed to 18.25°. The dispositions of the angles during COVID era (2019–2021) are shown in Fig. 20.

Three of the four cancers for which screening is highly effective and available rise to the top of reported cancers. Breast cancer is very close to these as well. These three cancers range from 55.49° to 43.53°. This angle of depression (11.96°) is twice that of the other cancers combined (6.29°). This means the other cancers were tightly grouped in their incidence. Look closely at the radar graph that leukemia and melanoma are in the second half of the angles of depression. Observe how those changed during the pandemic era.

**Fig. 21:**
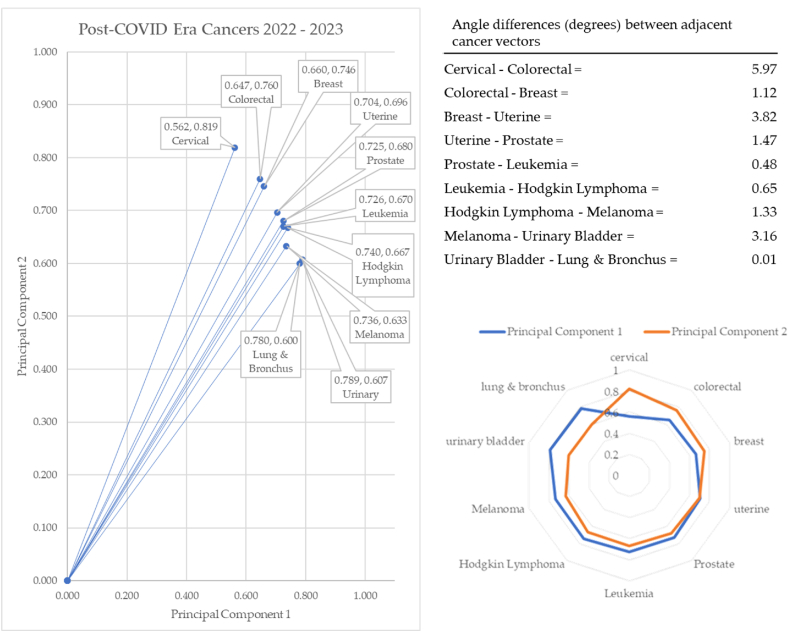
Dispositions in post-COVID era (2022–2023).

In the COVID-19 years of 2019–2021 (refer to Fig. 20), leukemia formed the vector with the most extreme angle (48.31°) and lung & bronchus cancer with the most conservative angle (41.09°). Between cancer vectors, the widest angle of depression was between leukemia and melanoma (4.28°) and the smallest angle of depression was between uterine and breast cancer (0.00°). The total angle vectors for the pre-COVID-19 era summed to 7.21°. This was 60% less than the pre-COVID-19 era. The dispositions of the angles post-COVID era (2022–2023) are shown in Fig. 21.

During the pandemic, several shifts occurred in the incidence of cancer reported. Leukemia and melanoma rose to the top. The other cancers are very closely aligned. The angle of depression between melanoma and lung & bronchus cancer is only 2.93°. Notice how differently this radar chart reports the data compared with both pre and post pandemic eras.

In the post-COVID-19 years of 2022–2023 (see Fig. 21), cervical cancer formed the vector with the most extreme angle (55.56°) and lung & bronchus cancer with the most conservative angle (37.55°). Between cancer vectors, the widest angle of depression was between cervical and colorectal cancer (5.97°) and the smallest angle of depression was between urinary and lung & bronchus cancer (0.01°). The total angle vectors for the pre-COVID-19 era summed to 5.16°. This was 72% less than the pre-COVID-19 era and 28% less than the COVID-19 era.

Several angles of depression between cancer triangles show dynamic movement over time. The cervical–prostate angle of depression moves from a minimum angle of −13.80°–16.11°. Also, the cervical-melanoma angle of depression moves from a minimum of −23°–20.15°.

**Fig. 22:**
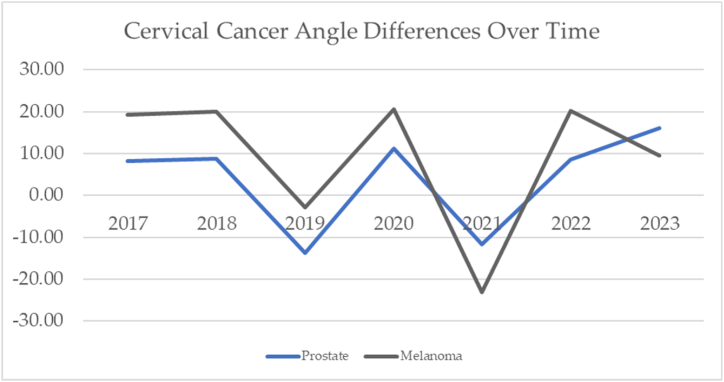
Cervical cancer mapped over time with prostate cancer and melanoma.

The cervical cancer angle differences are mapped over time with prostate cancer and melanoma in Fig. 22. These charts portray the change in angles over time. If there was no change in the incidence year to year, the slope would be zero.

These charts change in angles over time. It shows anomalies in the data for 2019 and 2021. If it were not for those two years, the lines would be flat (consistent change in incidence over time with a slope of zero).

In general, cervical cancer moves 23.11° over time, but the difference between 2017 and 2023 is only 0.72°. The years 2019 and 2021 are interesting to note. They do not follow the trend lines of the other years. These could have been adjustment years. More analysis should identify the reasons for these anomalies.

**Fig. 23:**
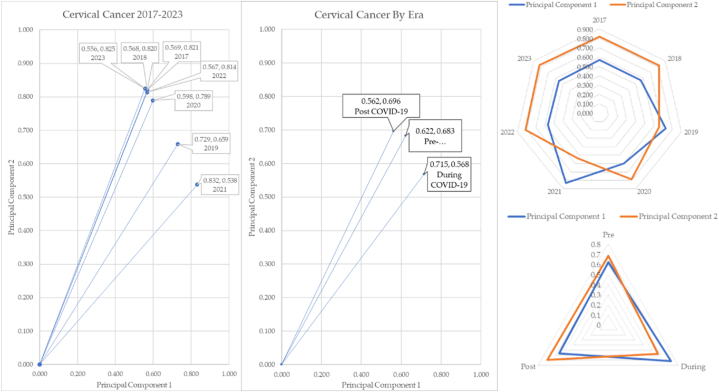
Cervical cancer over time and cervical cancer by COVID-19 eras.

When averaging the pre, during, and post- COVID-19 eras, there appears to be a shift in cervical cancer angle of 3.43°. The cervical cancer over time by COVID-19 and by pandemic eras is mapped in Fig. 23. We chose to map each cancer over the seven-year era and compare it with that cancer by era. The shift in depression angles was significant for several cancers.

The vectors change slightly when combined. Cervical cancer post pandemic has shifted upward by 3.43°. The radar chart shows how principal components 1 and 2 cross for the years 2019 and 2021. We saw this same trend when analyzing cervical cancer and melanoma over time. The years 2019 and 2021 are anomalies. The radar chart for the eras shows principal components 1 and 2 cross in the pre to during and during to post COVID-19. During COVID-19, the reporting of the incidence of cervical cancer was down by 9.2°.

**Fig. 24:**
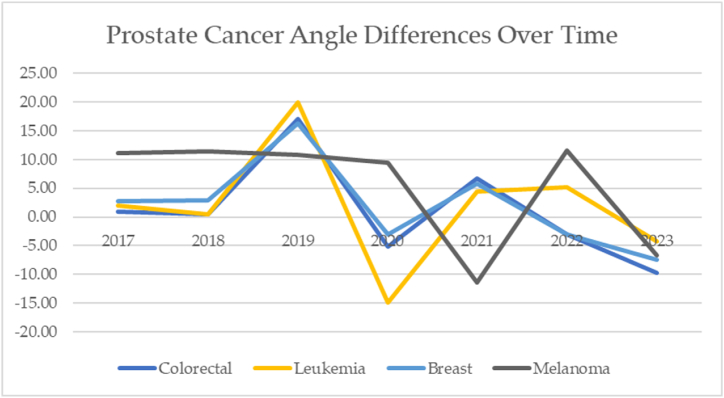
Prostate cancer angles of depression over time.

The prostate cancer angles of depression over time are mapped in Fig. 24. If the incidence of disease were constant, the slope would be zero.

The prostate-melanoma angle of depression moved across a range of −11.37 to 11.52° over time. The prostate-leukemia angle of depression moved across a range of −14.89 to 19.92°. The prostate-colorectal angle of depression moved across a range of −75 to 17.09. The prostate-breast angle of depression moved across a range of −7.5 to 16.30°. Prostate cancer angles of depression move across a range of 16.02°. When eras are compared, the pre-post-COVID-19 angle of depression is −6.80°.

**Fig. 25:**
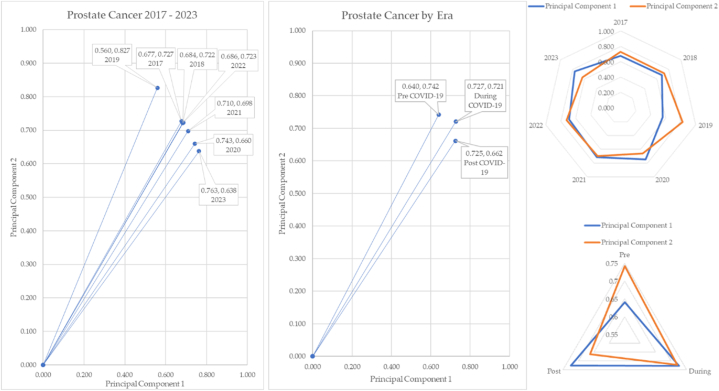
Prostate cancer over time and by COVID-19 eras.

The prostate cancer over time in COVID-19 eras is mapped in Fig. 25.

The vectors of prostate cancer change over time. Post pandemic, the incidence of prostate cancer decreases, and the angle of depression is 6.80°. As depicted by the radar charts, the principal components 1 and 2 cross several times. The radar chart for the eras is particularly unusual. Principal components 1 and 2 converge during the pandemic but are significantly different between pre and post.

**Fig. 26:**
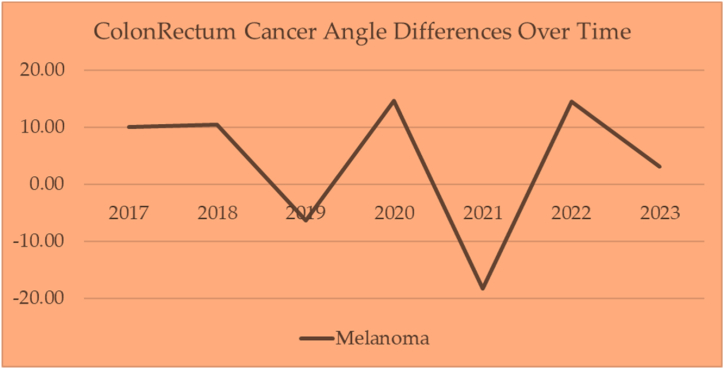
Colorectal cancer to melanoma angle of depression over time.

The colorectal-melanoma angle of depression moves from −18.15° to 14.59°. The colorectal cancer to melanoma angle over time is mapped in Fig. 26.

The colorectal-melanoma angle of depression moves from −18.15° to 14.59°. Overall, colorectal cancer only moves across a range of 11.89° over time, but when COVID-19 eras are compared, there is 4.26° shift in the angle of depression between pre- and post-pandemic.

**Fig. 27:**
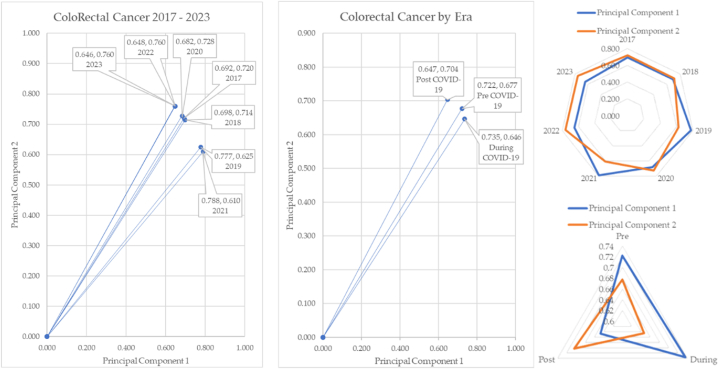
Colorectal cancer over time and by COVID-19 eras.

The colorectal cancer over time in COVID-19 eras is sketched in Fig. 27.

Colorectal cancer demonstrates another highly unusual shift in incidence from pre to post pandemic. The far-left chart of Fig. 27 demonstrates the changing angle of depression over the years we collected and analyzed data. The middle chart shows the shift of the aggregated data. Post pandemic incidence is higher than pre or during. The angle between pre and post pandemic is 4.26°. The radar charts show the unusual data shifts in 2019 and 2021, similar to what was observed in prostate and cervical cancers. Finally, the radar chart to the bottom right shows the shift when principal components 1 and 2 cross.

Hodgkin Lymphoma was static over time. Its angle of depression over time only moved across 4.62°. When eras were compared, the pre-post-COVID-19 era angle of depression measured only 1.29°.

**Fig. 28:**
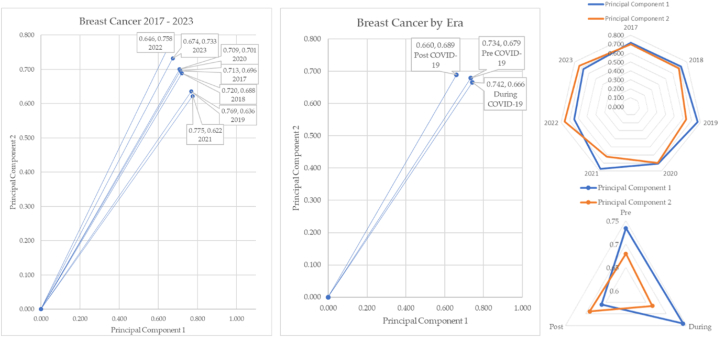
Breast cancer over time and by COVID-19 eras.

The breast cancer over time and in COVID-19 eras is sketched in Fig. 28.

Breast cancer over time illustrates unusual shifts in incidence, as seen in prostate, colorectal, and cervical cancers. The post-pandemic era shows an increase in incidence reported over the pre-pandemic data. This angle of depression is 3.47°. The radar charts show principal components 1 and 2 cross in the year 2021. The bottom right radar chart shows principal components 1 and 2 crossing which means that there is a significant change in how the incidence reacts in the post COVID-19 era.

**Fig. 29:**
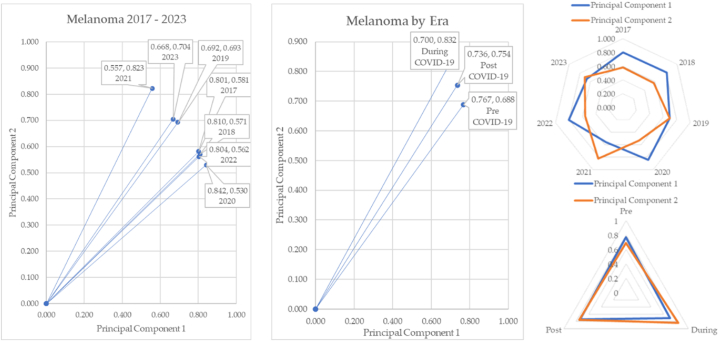
Melanoma over time and by COVID-19 eras.

Melanoma over time and in COVID-19 eras is sketched in Fig. 29.

Melanoma over time shows gradual shifts of the angles of depression. The during-COVID-19 years show a significant increase in incidence (8.06°), and the pre-to-post shows a smaller increase (3.82°). The radar charts show principal components 1 and 2 cross multiple times at years 2021 and 2013. The radar chart for the eras shows convergence of principal components 1 and 2 in the post-pandemic era.

All cancers are illustrated with radar charts for the pre, during, and post-pandemic eras.

Of interest in these radar charts is the cancer shift between eras. Cervical, colorectal, breast, and prostate cancers are consistently in the top 5 in the pre- and post-pandemic eras. In these two eras, principal components 1 and 2 cross at the third or fourth incidence cancer, respectively.

Furthermore, we computed an index ${\hat{M}}_{mode}=x_{\min}+\left(x_{\max}-x_{\min}\right){\hat{u}}_{\text{mode}}$ and track to address the pulls and pushes on the cancer incidences by the pandemic COVID-19 in three pre, during, and post eras. The most likely cancer incidences over the pre-, during, and post-COVID-19 eras are sketched in Fig. 31 below.

In general (that is considering all cancers), the projected most likely cancer incidences are sketched in Fig. 29 to notice that the pattern in the years 2017 and 2018, in the years 2019, 2020, 2021, versus in the years 2022, 2023 form distinct clusters. In specific, the breast cancer incidences happened to be slightly less during COVID-19 era (2019, 2020, and 2021) but more again in the post era (2022 and 2023). The less incidence during COVID-19 era might have been due to delaying or neglecting admitting or treating the breast cancer cases. The prostate cancer incidences seem to have hyped up in the end of COVID-19 and post-COVID-19 eras. All other cancer incidences were stable throughout the era 2017–2023.

Recall that the parameter estimates are ${\hat{\sigma }}^{2}=\ln\left(1+\frac{S_{x}^{2}}{\bar{x}}\right)$ and $\hat{\mu }=\ln\left(\bar{x}\right)-\frac{1}{2}\ln\left(1+\frac{S_{x}^{2}}{\bar{x}}\right)$, where $S_{x}^{2}$ and $\bar{x}$ are the data dispersion and average, respectively. Using the parameter estimates, we could examine the exceedance probability, $S\left(t\right)=\mathrm{Pr}\left[x\geq t\right]=\bar{\Phi }\left(\frac{\ln\;t-\hat{\mu }}{\hat{\sigma }}\right)$ with t arbitrarily chosen to be 1000 without any bias or reason for incidence of every cancer type. The exceedance probability to exceed 1000 cancer incidence is calculated, compiled, and displayed in Fig. 32. Except in the year 2020 (which was the middle of COVID-19 pandemic era), the exceeding probability was stable at 18%, 4%, 14%, 10%, 10%, 18%, 12%, 11%, 18%, and 12% for the breast, cervical, colorectal, uterine corpus, leukemia, lung & Bronchus, melanoma, Hodgkin lymphoma, prostate, and urinary cancer incidence. Interestingly, only in the year 2020, the exceeding probability to be over 1000 was elevated to 36%, 28%, 34%, 32%, 32%, 36%, 34%, 33%, 35%, and 33% for the breast, cervical, colorectal, uterine corpus, leukemia, lung & Bronchus, melanoma, Hodgkin lymphoma, prostate, and urinary cancer incidence.

**Fig. 30:**
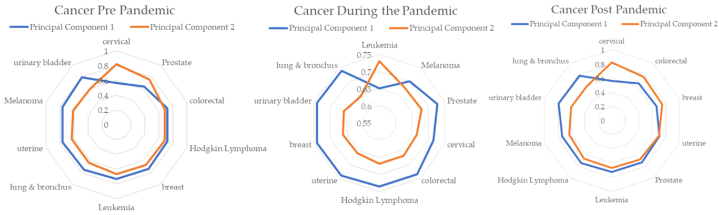
All cancers mapped over pandemic eras.

The proximity of the years with respect to the cancer incidences is identified and displayed in Fig. 30. The pre-COVID years 2017, 2018 and post-COVID years 2022, 2023 are in a cluster. During the COVID-19 years 2019, 2020, and 2021 are in turmoil with respect to cancer incidences. The proximity or far distance of years after marginalizing on all cancers is depicted in Fig. 33. Note that the pre- and post-COVID-19 eras are in closeness. The years during COVID-19 era are in turmoil. We chose to calculate hazard rates for further analysis.

**Fig. 31:**
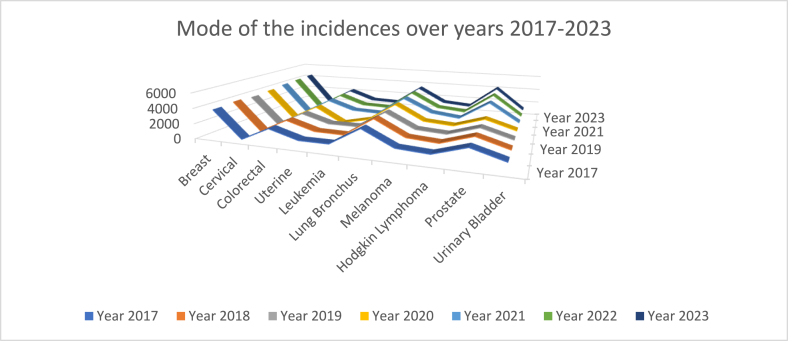
Most likely cancer incidences ${\hat{M}}_{\text{most}}$ over the years 2017–2023.

**Fig. 32:**
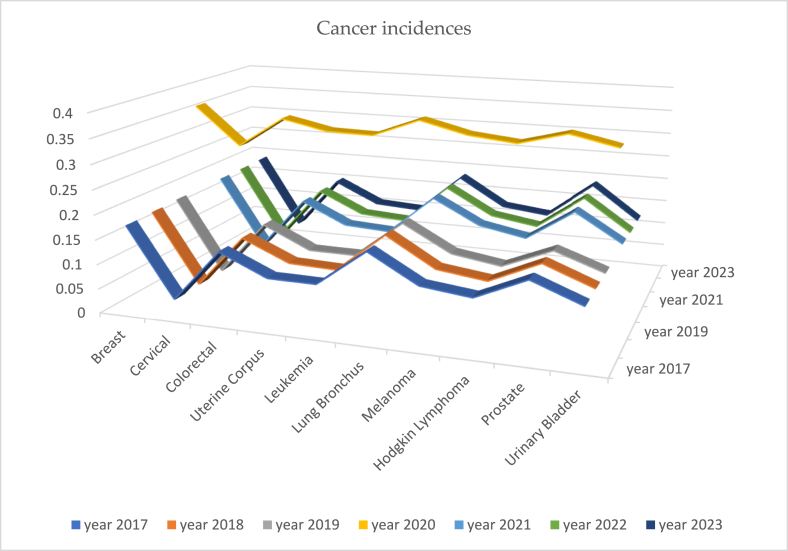
Was COVID-19 conducive or adversarial to cancer incidences.?.

**Fig. 33:**
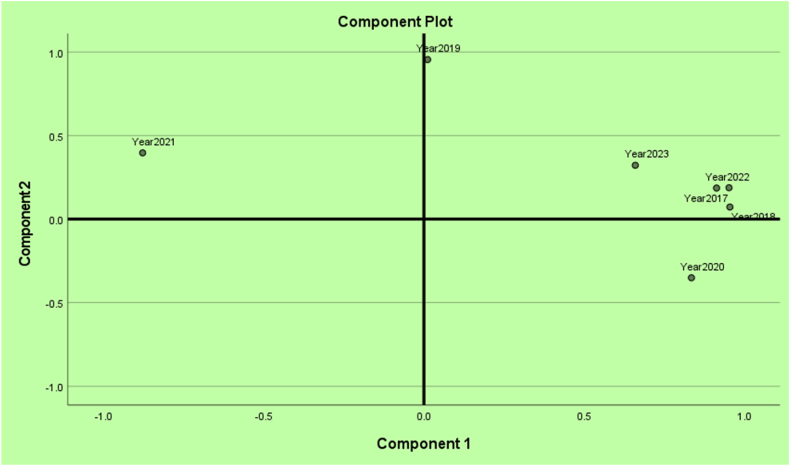
Data emits evidence for the proximity of cancer incidences over years.

**Fig. 34:**
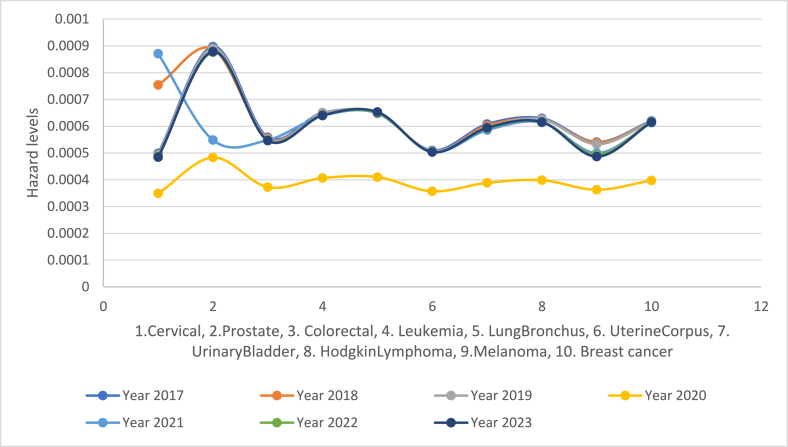
Hazard function for the cancer incidences during 2017 through 2023.

The hazard rates are displayed in Fig. 34. The hazard rates show how conducive COVID-19 was to cancer occurrence. The configurations are indicative of how many more cases (per 10,000 persons) of a particular type could occur in the year if they have survived to that year. The year 2020 is an anomaly in the sense that the hazard rate is flat. Although the pandemic did not start until the very end of 2019, the effects of COVID-19 were starting to affect the data. The hazard rate comes back to resemble like the pre- COVID-19 era of years 2017 and 2018. The year 2021 exhibits a puzzling mark of more breast cancer rate, making us to wonder whether this shift reflects resuming the admission or treatment of breast cancer patients which were delayed or avoided due to COVID-19. The hazard rates in the post-COVID-19 era of the years 2022 and 2023 had an unusual hyper spike.

**Fig. 35:**
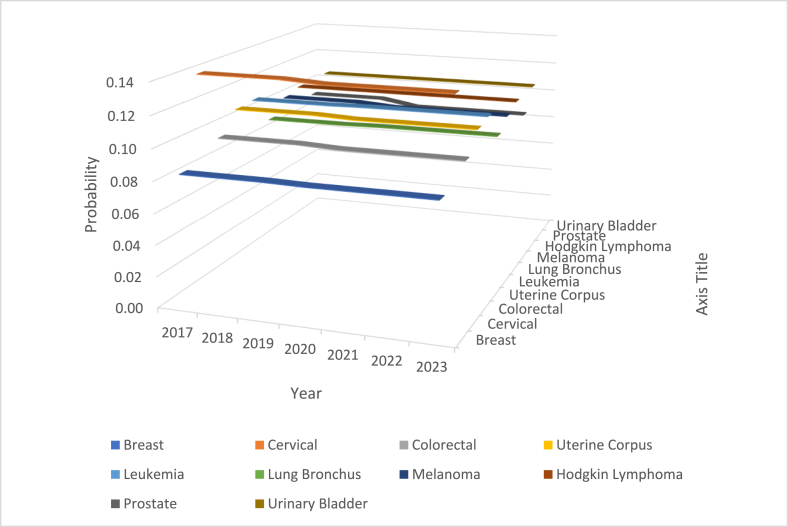
The Exceedance probability for each cancer during the years 2017 through 2023.

The exceedance probability for a cancer type to have over than 1000 per year among the breast, cervical, colorectal, uterine corpus, leukemia, lung & Bronchus, melanoma, Hodgkin lymphoma, prostate, and urinary cancers in pre-COVID, during COVID, and post-COVID years are displayed in Fig. 35. In every cancer type, the exceedance probability has increased. The breast cancer had a significant spike.

**Fig. 36:**
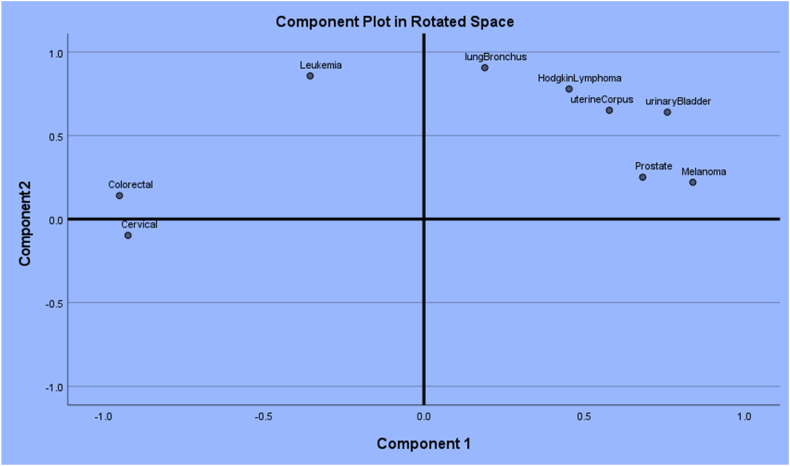
Proximities among cancers.

The proximity among the cancers breast, cervical, colorectal, uterine corpus, leukemia, lung & Bronchus, melanoma, Hodgkin lymphoma, prostate, and urinary tract cancers in the holistic sense are indicated in Fig. 36. The observed cancer incidences are correlated. That is, the vectors of cancer incidences in terms of years are non-orthogonal in a Euclidean space. The principal component analysis (PCA) is a multivariate technique which orthogonalizes correlated vectors. The values in the PCA are the basis on which the contents of the manuscript have been built and this approach is a novelty since no one has tried this way. As already mentioned, this article has demonstrated the novelty of utilizing the PCAs and makes useful interpretations of the analytic results to discern how much COVID has impacted the cancer incidences in US. The prostate and melanoma are in closer proximity. The uterine corpus, lung & Bronchus, Hodgkin lymphoma, and urinary cancer incidences were comparable. Cervical and colorectal cancers are in closer proximity. Leukemia cancer incidences are uniquely aloof. The authors of this article are not practicing medical professionals and hence, could not attribute any rationale for the phenomena.

**Fig. 37:**
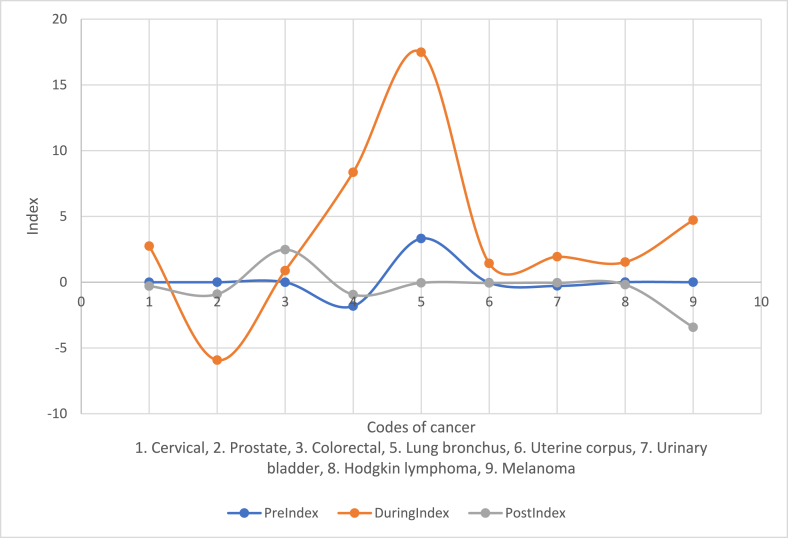
Indices from breast cancer vantage point of view.

Finally, we have created indices for other cancers from the vantage point of view at breast cancer using the angles. When some cancers happened to have inclination of negative angles from the breast cancer vantage point, the cancers fall in the 3rd or 4th quadrant of Euclidean two-dimensional graph. Consequently, the mean angle turns out to be negative with positive variances always. The estimates must be appropriately adjusted as follows for the sake of computing log values. Note that after the adjustments, ${\hat{\sigma }}_{angle}^{2}={\bar{x}}_{angle}\;\ln\left(1+\frac{s_{angle}^{2}}{\left|{\bar{x}}_{angle}\right|}\right)/\left|{\bar{x}}_{angle}\right|$ and ${\hat{\mu }}_{angle}={\bar{x}}_{angle}\;\ln\left(\left|{\bar{x}}_{angle}\right|\right)/\left|{\bar{x}}_{angle}\right|-0.5{\hat{\sigma }}_{angle}^{2}$. For example, the scale parameter of the pre-COVID-19 era for the cervical cancer incidence's angle from the breast cancer incidence's vantage point of view is estimated to be - 0.017 because the ${\bar{x}}_{angle}$ happened to be negative. The minus sign is indicative of smaller. The location parameter of the pre-COVID-19 era for the cervical cancer incidence's angle from the breast cancer incidence's vantage point of view is estimated to be 2.518 using the above formula. The shift in the cancer incidences is easily captured and explained by an index. The index for each cancer (with an appropriate sign) is defined and computed using ${Index}_{angle}=\frac{{\hat{\mu }}_{angle}}{\left({\hat{\mu }}_{angle}+{\hat{\sigma }}_{angle}^{2}\right)}$. For the pre-COVID-19 era, the cervical cancer incidence's angle index from the breast cancer incidence's vantage point of view is calculated to be – 0.007. For a comparison, the same cervical cancer incidence's angle index from the breast cancer incidence's vantage point of view during the COVID-19 era and in the post-COVID-19 era are 2.738 and – 0.298, respectively. These swinging values of the index portray that cervical cancer incidences are dynamic from the breast cancer incidence's vantage point of view. To capture and comprehend the dynamic and volatility of each cancer incidence, these indices are compiled and sketched in Fig. 37. Similar vantage points of view at a fixed location of each cancer in pre, during, and post-COVID-19 eras are feasible and routine. We skip them for the sake of briefness's sake. Notice that among the three eras, the most volatile is during COVID-19 era, according to Fig. 34.

## 4 Discussions

The orthodox method of analysis of breast, cervical, colorectal, uterine corpus, leukemia, lung & bronchus, melanoma, Hodgkin lymphoma, prostate, and urinary cancer incidences in the years 2017 through 2023 would have revealed which cancer had a significant occurrence. In our nontraditional but innovative approach, we were able to identify the closer proximities among the cancer types. Also, the probability for a specific cancer to have incidences exceeding 10,000 per year was calculable and comparable with both in pre-, during, as well as post-pandemic era. The hazard for each cancer to occur in pre-, during, as well as post-pandemic era could be assessed and compared. We can only hypothesize a reason for the shifts, and it could be a combination of factors. During the COVID-19 pandemic, there was limited access to healthcare, and therefore the healthcare screening that would normally detect cancer early was delayed. The findings in this article appear to be consistent with [46]. Cancer case trends following the onset of the COVID-19 pandemic: A community-based observational study with extended follow-up. The data in this study demonstrates a substantial reduction in cancer diagnoses following the onset of COVID-19, which appear to reach expected pre-COVID norms 12 months later. The largest reduction was noted among cancers that are typically screen-detected or identified as part of a routine wellness examination. This may have created a surge of incidence post-pandemic when screening services were offered again. In this cross-sectional study including 48,378 individuals with cancer diagnoses, a significant decrease in cancer diagnosis incidence was observed in the first few months of the pandemic, particularly in breast, colon, and rectal cancer incidence. Other cancer sites showed minimal long-term changes in incidence [47]. The findings of this article confirm what was speculated in Ref. [48] by its authors. The authors mentioned that the pandemic COVID-19 might have disrupted generally healthcare and in particular diagnosis of new cancer cases in a warning to the health professionals and public to strategize to identify and rectify missed diagnosis of cancer cases.

We do not see shifts across all cancers, however. We see it the most in breast, cervical, prostate, and colorectal cancers for which screening is highly screening that was limited during the limited access to healthcare services during the pandemic). We see the least amount of shift in Hodgkin Lymphoma: a cancer for which there is no screening. It is possible that the latter is so devastating to the body's lymphatic system, that the pandemic conditions had no effect. We may not have identified a large shift in melanoma because so much of that cancer can be diagnosed through telemedicine.

Another possibility is that there is no actual difference in the incidence of cancer. We may only see a change in cancer reporting. This could be due to a change in reimbursement or incentives.

Preventive measures such as social distancing, face masking, sanitations etc. would have certainly made a significant difference in the spread of COVID-19. Did those preventive measures have any shadow effect on the incidences of cancer? Did the preventive measures remain conducive to increasing the cancer incidences or as adverse to the cancer incidences? Future researchers might consider collecting and involving data on preventive measures to build regression curves and examine their strength of influence. This research direction might be more informative in helping public health experts and medical professionals contend with any future pandemics after drawing lessons learned from the COVID-19 era.

There might be comorbid effect of COVID-19 on cancers. The symptoms of cancer of any type set the stage for more vulnerability for a person to contract COVID-19 virus. Likewise, the immunity level of COVID-19 patients might have been compromised and hence, could become a cancer patient due to any environmental, genetic, exposure to carcinogenic agents. Vaccine communication efforts targeting populations affected by cancer and COVID-19 could form strategies that consider factors such as social norms, risk perceptions, and trust to enable herd immunization. Though some segments of the population hesitate to accept the vaccination to safeguard from COVID-19, the public health professionals ought to prioritize the importance of vaccinations more among those who survived with any cancers. The positive test result in persons might expedite more cancer screening and vice versa. Several healthcare professionals contemplate that COVID-19 has constrained and impaired daily routines of life around the globe. The healthcare services in hospitals, clinics, and emergency divisions have prioritized treating COVID-19 patients by delaying or avoiding the routine screening or caring for the cancer patients. Some cancer patients would have survived longer with treatment without the pandemic COVID-19. The organs of the COVID-19 patients might have been too heavily infected to resist the cancerous agents. The COVID-19 virus might onset or accelerate cancers. In addition to the immediate life-threatening situation for the COVID-19 cases, the pandemic virus has a long-term effect due to harboring oncogenic viruses with mutational complications. In conclusion, Covid has stricken hard on cancer patients during Covid-era and post-Covid-era [49]. Our research focus was to assert whether the pandemic COVID-19 has shifted the cancer incidences within pre, during, and post era. This focused research itself happened to be quite extensive. Of course, the analytic results are stochastic. Devising a statistical methodology to check whether an angle or shift in the result can be judged for its statistical significance is going to be very involving and hence, falls outside the scope of this current research. Nevertheless, it is worth investigating later by us in another manuscript. We believe that our line of research thinking would be extended by future researchers. With this thought, we have placed the data in a repository of our university and inscribed a link to access the data. The source of the original data is https://www.cancer.org/research/cancer-facts-statistics/all-cancer-facts-figures. In conclusion, we mention a few limitations of our work and results.

## Limitations

The primary limitation to this research is inherent to PCA itself. In general, there is low interpretability of principal components, which are linear combinations of features from original data. A second limitation is the information loss inherent to the calculation process (Keboola.com. https://www.keboola.com/blog/pca-machine-learning Accessed on January 2, 2024).

## Future research

Future research should examine a longer period post-pandemic to see if vectors returned to pre-pandemic levels. This research would highlight short-term effects of treatment delay. However, if this research shows a consistency of different vectors post-pandemic, then it should trigger additional studies to investigate why.

## Data availability

The data were downloaded from the American Cancer Society https://www.cancer.org/research/cancer-facts-statistics/all-cancer-facts-figures [2]. Researchers could access the data in a repository via the link https://doi.org/10.18738/T8/MXRXSX.

## Credit author statement

All authors contributed equally in motivation, conceptualization, data analysis, interpretations, etc.

## Declaration of competing interest

The authors declare that they have no known competing financial interests or personal relationships that could have appeared to influence the work reported in this paper.
