# Supplementary material for: The effect of COVID-19 on cancer incidences in the U.S
Source: Heliyon. 2024 Mar 30;10(7):e28804. doi: 10.1016/j.heliyon.2024.e28804 (PMC11004761; doi:10.1016/j.heliyon.2024.e28804)
Supplement: Multimedia component 1 [file mmc1.docx]

**Supplementary Materials**

2/24/2024

Dear Editor of Heliyon,

These supplementary materials are on the manuscript with the title:

**The Effect of COVID-19 on Cancer Incidences in the U.S.**

Ramalingam Shanmugam^1^, Larry Fulton^2^, C. Scott Kruse^1^, Brad Beauvais^1^, Jose Betancourt^1^, Gerardo Pacheco^1^, Rohit Pradhan^1^, Keya Sen ^1^, Zo Ramamonjiarivelo^1^, Arvind Sharma^2^

^1^ Texas State University, School of Health Administration, Encino Hall, Room 250A, 601 University Drive, San Marcos, TX 78666

^2^ Boston College, Woods College of Advancing Studies, St. Mary’s Hall South, Chestnut Hill, MA 02467.

1. We installed the data in a repository. Researchers can access the data in a repository via the link <https://doi.org/10.18738/T8/MXRXSX>.
2. We added the ‘Data availability statement’ section in the manuscript right before the ‘References’ section. We have resubmitted the revised version in <https://www.editorialmanager.com/>heliyon/ in Author’s menu 'Submission Needing Revision'.
3. We chose all applicable statements in ethics declarations list (from <https://www.cell.com/heliyon/ethics>). To be specific,

**Declaration of interests**
☒ The authors declare that they have no known competing financial interests or personal relationships that could have appeared to influence the work reported in this paper.
☐The authors declare the following financial interests/personal relationships which may be considered as potential competing interests.

| **Reviewer 4 comments** | **Authors response** |
| --- | --- |
| Please add ‘Data availability statement’ section right before the ‘References’ section. | The data were downloaded from the American Cancer Society [1].   1. <https://www.cancer.org/research/cancer-facts-statistics/all-cancer-facts-figures>.   Researchers can access the data in a repository via the link <https://doi.org/10.18738/T8/MXRXSX> |
| Please choose all applicable statements in our ethics declarations list (available here: https://www.cell.com/heliyon/ethics) and include them as a complete ethics statement in the declarations section at the end of your manuscript. | From <https://www.cell.com/heliyon/ethics>.  **Declaration of interests**  ☒ The authors declare that they have no known competing financial interests or personal relationships that could have appeared to influence the work reported in this paper.  ☒ The authors declare the following financial interests/personal relationships which may be considered as potential competing interests. |

Thank you and best regards,

Ram

**Ram Shanmugam, Ph. D., M. S. (Math Stat and Econometrics), M. S. (OR), FASA, EFISI**

**Honorary Professor, School of Health Administration,**

**Texas State University, San Marcos, TX 78666, USA**

**Office phone: +1 512 245 9772, Email** [**rs25@txstate.edu**](mailto:rs25@txstate.edu)**, Fax +1 512 245 8712**
